# TGFβ1-Induced *Baf60c* Regulates both Smooth Muscle Cell Commitment and Quiescence

**DOI:** 10.1371/journal.pone.0047629

**Published:** 2012-10-26

**Authors:** Abhishek Sohni, Francesca Mulas, Fulvia Ferrazzi, Aernout Luttun, Riccardo Bellazzi, Danny Huylebroeck, Stephen C. Ekker, Catherine M. Verfaillie

**Affiliations:** 1 Stem Cell Institute, Department of Development and Regeneration, K.U.Leuven, Leuven, Belgium; 2 Genetics Cell and Developmental Biology, University of Minnesota, Minneapolis, Minnesota, United States of America; 3 Center for Tissue Engineering, University of Pavia, Pavia, Italy; 4 Dipartimento di Informatica e Sistemistica, University of Pavia, Pavia, Italy; 5 Center for Molecular and Vascular Biology, K.U.Leuven, Leuven, Belgium; 6 Laboratory of Molecular Biology (Celgen), Department of Development and Regeneration, K.U.Leuven, Leuven, Belgium; 7 Department of Biochemistry and Molecular Biology, Mayo Clinic, Rochester, Minnesota, United States of America; William Harvey Research Institute, Barts and The London School of Medicine and Dentistry, Queen Mary University of London, United Kingdom

## Abstract

Smooth muscle cells (SMCs) play critical roles in a number of diseases; however, the molecular mechanism underlying their development is unclear. Although the role of TGFβ1 signaling in SMC development is well established, the downstream molecular signals are not fully understood. We used several rat multipotent adult progenitor cell ((r)MAPC) lines that express levels of *Oct4* mRNA similar to hypoblast stem cells (HypoSC), and can differentiate robustly to mesodermal and endodermal cell types. TGFβ1 alone, or with PDGF-BB, induces differentiation of rMAPCs to SMCs, which expressed structural SMC proteins, including α-smooth muscle actin (αSMA), and contribute to the SMC coat of blood vessels in vivo. A genome-wide time-course transcriptome analysis revealed that transcripts of *Baf60c*, part of the SWI/SNF actin binding chromatin remodeling complex D-3 (SMARCD3/BAF60c), were significantly induced during MAPC-SMC differentiation. We demonstrated that BAF60c is a necessary co-regulator of TGFβ1 mediated induction of SMC genes. Knock-down of *Baf60c* decreased SMC gene expression in rMAPCs whereas ectopic expression of *Baf60c* was sufficient to commit rMAPCs to SMCs in the absence of exogenous cytokines. TGFβ1 activates *Baf60c* via the direct binding of SMAD2/3 complexes to the *Baf60c* promoter region. Chromatin- and co-immunoprecipitation studies demonstrated that regulation of SMC genes by BAF60c is mediated via interaction with SRF binding CArG box-containing promoter elements in SMC genes. We noted that compared with TGFβ1, Baf60c overexpression in rMAPC yielded SMC with a more immature phenotype. Similarly, *Baf60c* induced an immature phenotype in rat aortic SMCs marked by increased cell proliferation and decreased contractile marker expression. Thus, Baf60c is important for TGFβ-mediated commitment of primitive stem cells (rMAPCs) to SMCs and is associated with induction of a proliferative state of quiescent SMCs. The MAPC-SMC differentiation system may be useful for identification of additional critical (co-)regulators of SMC development.

## Introduction

Smooth muscle cells (SMCs) are a unique class of muscle cells that have diverse developmental origins. Unlike cardiac or skeletal muscle, SMCs can develop from splanchnic mesoderm [1], neural crest cells [1], [2] local mesenchyme [1], [3] and perhaps endothelial cells [4]. However, little is known regarding the molecular mechanisms underlying SMC specification and differentiation during development. Dysfunction of SMCs forms the basis for a number of diseases, including the formation of aneurysms, and atherosclerosis or asthma onset [3], [5], [6]. The molecular mechanisms forming the basis for the decrease or increase in SMC formation underlying these disorders are also not well understood.

Developmentally, SMC induction has been attributed to Transforming Growth Factor type beta (TGFβ) signaling. Disruption of TGFβ1 signaling in mice due to inactivating mutations either in *TGFβ1* [7], *Alk1* [8], [9], *Endoglin* [10] or *Smad5* [11] causes embryonic lethality attributed to vascular defects in the developing embryo and extra-embryonal tissue. This embryonic lethality has precluded the use of ubiquitous knock-out approaches in the analysis of the molecular mechanism involved in TGFβ1 as well as of other growth factor or signaling pathways involved in SMC development (reviewed by Owens *et al.*) [3]. In addition to conditional knock-out mice, investigators have used *in vitro* cell models to investigate the mechanisms underlying SMC specification and differentiation [12], [13]. Similar to TGFβ1, platelet derived growth factor-beta (PDGF-BB) has also been shown to be important for SMC proliferation [14] and migration during early embryogenesis [15].

We used MAPCs from rat and induced differentiation to SMCs as described by Ross *et al.* [16]. Compared with the study by Ross et al, the rMAPCs used in the current study expressed high levels of *Oct4* transcripts, at levels near those of Embryonic Stem Cell (ESC) [17], [18] whereas the rMAPC used by Ross *et al.* expressed no *Oct4*. Ulloa *et al.* demonstrated that the cells isolated under MAPC culture conditions that express low levels/no *Oct4* are more similar to mesenchymal stem cells whereas rMAPCs with *Oct4* expression levels near those of ESCs have a more primitive phenotype [17]. More recently, we have demonstrated that rMAPCs are highly similar to hypoblast stem cells (HypoSCs) derived from rat blastocysts. Moreoever, rMAPCs and rHypoSCs have considerable *in vitro* plasticity, as they generate definitive endoderm via an apparent primitive streak intermediary [19]. Differentiation of mesoderm committed stem cells such as MSC or rMAPC clones lacking *Oct4* expression, would not reveal early commitment genes. As we wished to discern signal pathways required to commit primitive stem cells to SMCs, we here adapted the protocol used by Ross *et al.* to rMAPCs expressing high levels of *Oct4*. We confirm that rMAPCs can be committed to SMCs that can functionally contribute to the smooth muscle cell coat of vessels *in vivo*. Transcriptome analysis of the differentiation process demonstrated that the transcript encoding *Baf60c* (also named *Smarcd3*) of the SWI/SNF actin binding chromatin remodeling complex D-3 is upregulated during SMC differentiation. We found that BAF60c is necessary and may be sufficient to specify rMAPCs to SMCs and interacts with SRF to co-regulate SMC genes via CArG box elements. Furthermore, the expression of *Baf60c* is regulated via the SMAD2/3 dependent TGFβ1 pathway. Finally, we demonstrate that *Baf60c* is important in inducing a proliferative state in mature SMCs.

## Materials and Methods

### Cell culture and differentiation

Cell lines used include the previously described lines rMAPC-1 and Cl19 [17], and newly isolated rMAPC lines 3c3 and KS1 [18]. Undifferentiated rMAPCs were cultured as previously described [17], [18]. To induce SMC differentiation, cells were plated at 1500 cells per sq. cm. in MAPC basal medium [17], [18] supplemented with TGFβ1 (2.5 ng/ml) (R&D systems) and PDGF-BB (5 ng/ml) (R&D systems) in the absence of FBS (unless otherwise specified) at 37°C, 5% CO_2_ and 5% O_2_. Complete medium changes were done every two days for 6 days. From day 6 onwards, cells were cultured in basal rMAPC medium with 10% FCS (HyClone laboratories, UT, USA) (expansion media). *Baf60c*-transduced rMAPC were differentiated in basal rMAPC medium without growth factors or FCS, after seeding at 2000–3000 cells per sq. cm for 9 days. Primary rat aortic smooth muscle cells (RAOSMCs) were cultured in DMEM F12 medium (both from Lonza, Basel, Switzerland) containing 20% heat inactivated FBS (Hyclone) at 37°C in 5% CO_2_ and 20% O_2_.

### RNA isolation, cDNA synthesis and RT-q-PCR

Cells were harvested using RLT lysis buffer (Qiagen, CA, USA) supplemented with β-mercaptoethanol (Sigma-Aldrich). The lysed cells were stored at −80°C until the RNA extraction. RNA isolation was performed using the microRNA isolation kit (Qiagen) following manufacturer's instructions with on-column DNase treatment. The RNA was quantified and 1 µg was used for cDNA synthesis using the Superscript III reverse transcriptase kit (Invitrogen, CA, USA). Realtime PCR was performed using the SYBR®green method (Invitrogen) on an Eppendorff realtime apparatus (Eppendorff, NY, USA). Gene expression was quantified using GAPDH as internal control using the ΔCt method. In some cases, we describe gene expression as fold change compared with day 0. The gene specific primers used are listed in Supporting Information S1.

### Immunostaining of in vitro differentiated rMAPCs

Cells and tissues were fixed in 10% NBF (neutral buffered formalin) (Sigma-Aldrich, MO, USA) and standard staining protocols were followed. RAOSMCs were used as positive control. The list and concentrations of antibodies used is provided in Supporting Information S2.

### Matrigel plug assay

A detailed protocol has been previously reported [20], [21] and is described in supplemental information (Supplemental Information S3). Briefly, rMAPCs were transduced with an eGFP-encoding retroviral vector. 1×10^6^ undifferentiated rMAPCs or rMAPC-derived SMCs were mixed in cytokine-reduced Matrigel (BD Biosciences, CA, USA) and injected under the skin of 8-week old athymic nude Foxn1 mice (Jackson Laboratory, ME, USA). 3 weeks later, plugs were harvested and processed for double staining with antibodies against αSMA and GFP. The total number of vessels (marked by αSMA staining) and vessels surrounded by GFP-αSMA double positive cells were counted. In addition, the total area of the section and area of DAPI staining were computed using Axiovision ver 4.8 software (Zeiss). Animal studies were approved by the Ethics Committee at KU Leuven.

### Microarray analysis

A detailed protocol of hybridization and analysis of data is described in supporting information (Supporting Information S3). Briefly, clones 3c3 and Cl19 were assessed at day 0, day 2, day 4 and day 6 of differentiation in triplicate along with rat primary smooth muscle cell RNA (Cell Applications, San Diego, CA, USA) as positive control in duplicate. RNA was isolated as described above. Sample processing and data normalization were performed by the VIB MicroArray Facility, KULeuven (www.microarrays.be). Differentially expressed probes were identified with a gene selection method [22] that relies on the calculation of the area of the region bounded by each gene expression time series and a control profile. For each probe, the control profile was taken as a constant profile with values equal to the expression at the first time point (d0). Differentially regulated probes were obtained with a significance level of 0.01.

### *Baf60c* knockdown

#### shRNA containing lentiviral vector

To generate an inducible silencing lentiviral vector, four *Baf60c* targeting shRNAs were generated and tested. The shRNAs were cloned into the pTRIPZ vector (Openbiosystem, AL, USA). In addition, a non-silencing pTRIPZ vector was purchased (Openbiosystem) [23]. Lentiviral vector was produced using the Lenti-X lentiviral expression system, according to the manufacturer's protocol (Clontech Laboratories, CA, USA). Transduction of rMAPCs was performed using an MOI of >10, using standard procedures. The transduced cells were selected for puromycine resistance (0.5 µg/ml) (Sigma-Aldrich, MO, USA). Among several *Baf60c*-targeting vectors tested, only one shRNA (Supporting Information S1) was able to provide significant knockdown of *Baf60c* expression upon doxycycline (Sigma-Aldrich) induction.

#### siRNAs

We purchased ON-target siRNA pool (Dharmacon, CO, USA), that specifically targets *Baf60c*. rMAPCs treated with TGFβ1 or RAOSMCs were transiently transfected with the *Baf60c* targeting pool or a non-targeting control siRNA pool using either Lipofectamine (Invitrogen, CA, USA) or PowerFect transfection reagent (SignaGen laboratories, MD, USA). Cells were harvested 24–48 h post transfection.

### Western blot

For Western blotting, cells were lysed in RIPA buffer (Sigma-Aldrich) with protease inhibitors (Roche Applied Sciences, Switzerland). Protein concentrations were quantified using the BCA kit (Thermo Scientific, IL, USA). Equal quantity of protein was run on 10% or 4–20% Tris-Glycine gels (Bio-Rad Laboratories, CA, USA) using 1×TGS buffer (Bio-Rad Laboratories). The proteins were blotted onto nitrocellulose membranes using a Bio-Rad gel transfer apparatus (Bio-Rad Laboratories) in tris-glycine buffer (Bio-Rad Laboratories). Membranes were blocked in 5% milk for 30–40 min and incubated with primary antibody for 1 hr to overnight, at 4°C. Secondary staining was done with an HRP-conjugated secondary antibody and protein bands were visualized using SuperSignal West Pico chemiluminescent substrate (Thermo Scientific, IL, USA). Quantification of the band intensities was performed using ImageJ software following NIH guidelines. Antibodies used are listed in Supporting Information S2.

### *Baf60c* expression vector

The pENTR-D Topo vector (Invitrogen) was used to generate entry vectors by directional cloning of GFP-T2A with *AgeI* and *ClaI* restriction sites following the T2A sequence. Rat *Baf60c* cDNA (purchased from Openbiosystem, AL, USA) was sub-cloned into the entry vector, without the stop codon and flanked by *AgeI* and *ClaI* restriction sites to generate the GFP-T2A-*Baf60c* fragment. The entry vectors (with GFP-T2A and with *Baf60c*) were then recombined into the destination vector pLENTI4-TO-V5–DEST (destination vector and expression kit from Invitrogen, CA, USA) using the gateway cloning protocol. The vector generates GFP-T2A-*Baf60c* (Fig. S1) or GFP-T2A mRNA, which upon translation generates separate proteins for GFP and BAF60c tagged by a V5 epitope. Tet-mediated regulation was obtained by co-transducing a vector expressing Tet-R (pLenti6-TR, Invitrogen). Lentiviral vector production was done using the ViraPower T-REx Lentiviral expression system (Invitrogen). Transduction of rMAPCs was performed using an MOI of 5 for Tet-R encoding lentiviral vector as per manufacturer's recommendations. The Tet-R transduced cells were selected using blasticidin at 2 µg/ml (Invitrogen). Subsequently, the rMAPCs were transduced with the Tet inducible *Baf60c* containing vector, at an MOI of >10, followed by selection with 100 µg/ml Zeocin (Invitrogen).

### Cell cycle analysis

RAOSMCs were transiently transfected with an anti-*Baf60c* pool or non-targeting pool of siRNAs, or overexpression vector(s) (GFP-T2A or GFP-T2A-*Baf60c*) were trypsinized and washed with PBS to remove any traces of media/cell debris. Cells were fixed in 70% ethanol in PBS at −20°C over night. The following day the cells were washed with PBS to remove traces of ethanol and treated with Hoechst (Invitrogen, CA, USA) for 15–30 min at 37°C. The cells were analyzed using the BD-FACS ARIA III (BD biosciences).

### TGFβ signaling inhibition

Cells were cultured in basal rMAPC media. Cells were seeded at 1500–3000 cells per sq. cm. and treated with either TGFβ1 (2.5 ng/ml) alone or combined with either the TGFβR inhibitor (SB431542) (Tocris Bioscience, MO, USA) (10 µg/ml) or SIS3 (8 µg/ml) (Sigma-Aldrich). Cells were harvested after 2 days in RLT lysis buffer for RNA extraction or in RIPA buffer for protein extraction.

### Luciferase assay

The promoter of *Baf60c* (2.3 kb upstream of the 5′ UTR) was cloned from genomic DNA of rMAPCs into the pGL3 vector with XhoI and Bcul restriction sites to drive firefly Luciferase expression and generate wild type (WT) vector. Whole plasmid PCR amplification of the WT plasmid was performed using the 5′ phosphorylated “mutant” primers [5′-(P)-CAcgttCAagctggCACACATGCACGAAC-3′ (clockwise/forward) and 5′–(P)- TTTccgTcctTaTGTGTGCATGTGTGTGT (anti-clockwise/reverse) (IDT-DNA Technology, IA, USA)] to generate deletion of the CAGA (Smad binding element - SBE) present between -2096 bp and -2150 bp of *Baf60c* promoter containing plasmid. The PCR amplification was performed using Phusion Hot Start High Fidelity Polymerase (NEB, MA, USA), with annealing performed at 48°C with 25 cycles and a cycle extension of 8 min. The PCR product was then gel purified and ligated overnight using T4 DNA ligase (Fermentas, Germany) to generate circular DNA. The ligated product was transformed into NEB-5α competent bacteria (NEB, MA, USA). rMAPCs were co-transected with 1 µg of WT or mutant promoter-reporter plasmid and 100 ng of Renilla luciferase plasmid (Promega, WI, USA) using FuGENE (Roche Applied Sciences) transfection reagent. As a positive control, the TGFβ-responsive CAGA element basal promoter driving luciferase [24] was used. Transfected rMAPCs were either untreated or treated with 10 µg/ml TGFβ1 for 48 h and luciferase activity was assessed using the dual-luciferase reporter assay system (Promega, WI, USA). The relative expression of firefly luciferase to renilla luciferase was compared between mutant and wild-type *Baf60c* promoter elements.

### Chromatin immunoprecipitation

BAF60c-V5 expressing clones of rMAPCs were treated with TGFβ1 for two days. Cells were trypsinized and washed with PBS. The TF ChIP kit (Diagenode, Belgium) was used to perform IP of the sheared chromatin. Cells were lysed following manufacturer's recommendations and were sonicated, using the Branson digital sonifier 250 (Branson Ultrasonics Corporation, CT, USA) with 9 cycles of 30% amplitude for 10 seconds. IP of chromatin bound by BAF60c was performed using agarose conjugated anti-V5 rabbit polyclonal antibody (Abcam, UK), or isotype control rabbit IgG (BD Biosciences) in equivalent concentrations. The IP and washes were performed following manufacturer's protocol (Diagenode). The immunoprecipitated DNA was subjected to qPCR using primers listed in Supporting Information S1.

### Co-immunoprecipitation

Buffers were prepared as previously described [25], [26]. RAOSMC cells were trypsin-harvested and washed with PBS. Pellets of cells were lysed in lysis buffer and incubated on ice for 30 min. Protein A beads (GE healthcare lifesciences, Belgium) were used for coupling of the antibody. The protein lysate was co-incubated with beads and antibody overnight at 4°C. The beads were washed three times and the complex eluted directly in sample loading buffer with dithiothreitol (DTT). The eluted samples were used for western blotting.

### Statistics

Comparisons were performed using Student's t-test or one-way ANOVA (Kruskal-Wallis test); p-values lower than 0.05 were considered significant.

## Results

### rMAPCs can be committed to SMCs that contribute to coating of vessels in an in vivo Matrigel plug assay

**Figure 1:**
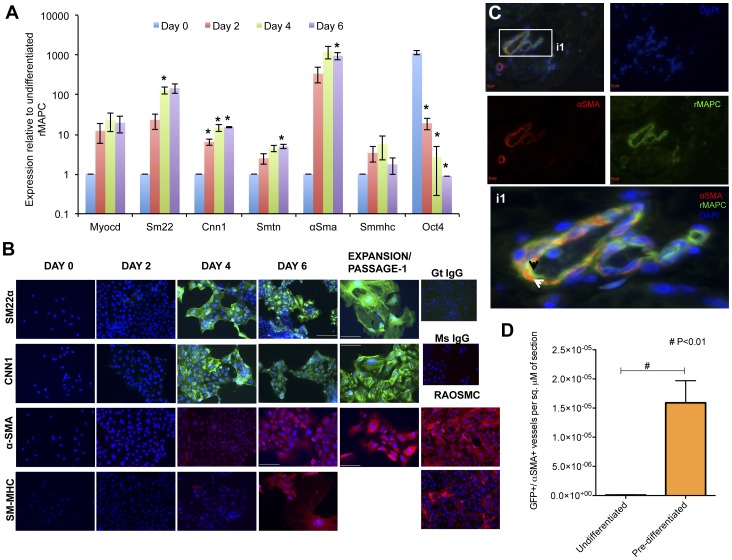
Differentiation of rMAPCs to smooth muscle cells. rMAPCs were cultured for 6 days with TGFβ1 and PDGF-BB in serum free medium. On day 6, cells were harvested and re-plated in serum containing medium (Fig. S6), without exogenous growth factors. **A**. Expression of smooth muscle genes in differentiation Cl19 rMAPC represented as expression relative to GAPDH (Mean±s.e.m of n = 3–5, p<0.05; data for other lines can be found in Fig. S5). **B**. Cells were fixed and stained on days 0, 2, 6 and after passage/expansion in serum containing media with antibodies against SM22α, CNN1, α-SMA and SM-MHC. Nuclei were identified using Hoechst or Dapi. Images at 40× magnification, scale bar 50 µM; representative example one of 3 clones. **C**. Undifferentiated MAPCs and MAPC-derived SMCs were implanted in Matrigel plugs supplemented with VEGF and FGF2 under the skin of nude mice, and the plugs were harvested after 21 days. A representative image of a vessel formed by smooth muscle (αSMA) derived from rMAPCs indicated by αSMA colocalized to the GFP cell (white arrow) in the inset i1. The endothelium contribution of the vessel may have been from (GFP) rMAPCs injected (black arrow). Scale bar 10 µM. **D**. Quantification of the number of vessels wherein GFP and αSMA colocalised, per sq µm of the section (Mean±s.e.m of n = 4; Student's t-test p<0.01).

TGFβ1 plays an important role in SMC development [7], [9], [10]. Moreover, TGFβ1 is important to induce SMC differentiation in mesoderm committed ESCs as well as adult stem cells, such as mesenchymal stem cells (MSCs) and MAPCs [12], [16], [21]. Previously, we demonstrated that rat MAPCs [16], [17] could be committed to SMC by addition of TGFβ1. The rMAPCs, used in that study did not express *Oct4* and were later shown to be similar to mesenchymal stem cells (rClone1 in Ulloa *et al.*) [17]. As we wanted to define signaling pathways responsible for TGFβ1-mediated SMC differentiation from even more primitive cells, we here used four individually isolated rMAPC lines that express high levels of *Oct4* and also generate in a robust manner endodermal progeny aside from mesodermal progeny, to generate functional SMC-like cells [17], [18]. Similar to the studies from Ross *et al.* with MAPC not expressing *Oct4* [16], rat MAPC expressing high levels of *Oct4* can also be committed to cells with transcript and protein marker expression found in SMCs (Fig. 1A,B, S2, S3, S4). The differentiated cells expressed αSMA in a similar pattern as RAOSMCs and myosin heavy chain (SM-MHC) protein was present in 60% of cells on d6, in a pattern comparable to the expression in RAOSMC (Fig. 1B).

A Matrigel plug assay was performed to assess the ability of rMAPC-derived SMCs to contribute to vessel formation in vivo. Retrovirally transduced GFP-labeled rMAPCs and MAPC-derived SMCs were pre-mixed in Matrigel containing VEGF_165_ and FGF2 and injected under the dorsal skin of nude mice. After 3 weeks the plugs were harvested and analyzed macro- and microscopically (Figs. 1C and S5A, B). In Matrigel plugs loaded with undifferentiated rMAPCs we observed localization of GFP staining to endothelium (as previously reported [20], [21]) and/or contribution of GFP positive (+) cells to the SMC layer that surrounds the vessels (Fig. 1C). When rMAPC-derived SMCs were incorporated in the Matrigel plugs, vessels were surrounded by cells positive for both eGFP+/αSMA+ cells, consistent with the notion that the rMAPCs contribute actively to the generation of the SMC coat surrounding vessels (Fig. 1C). We calculated the ratio of double-positive GFP+/αSMA+ cells over the total number of vessels stained (αSMA+) in both conditions and found a 10.5-fold higher contribution of SMC-coating surrounding vessels by pre-differentiated rMAPC-derived SMCs (33.02±0.08) compared with undifferentiated rMAPCs (3.13±0.12) (Fig. S5B). Similarly, we found that this difference was significant (p<0.05) when normalized to the total tissue area (co-localized vessels/total surface of section) (Fig. 1D).

### Genome-wide transcriptome analysis of time-course of differentiation

**Figure 2:**
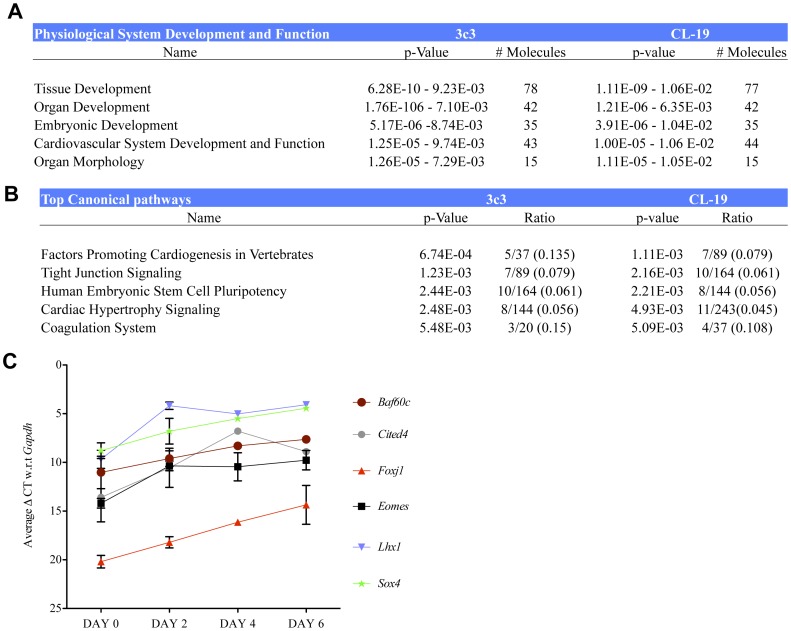
Genome wide transcriptome analysis of rMAPC-SMC differentiation. rMAPCs (Cl19 and 3c3) were cultured for 6 days with TGFβ1 and PDGF-BB in serum free medium, and RNA harvested from triplicate samples on days 0, 2, 4 and 6. In addition, RNA from cultured RAOSMCs was obtained in duplicate. **A**. Ingenuity pathway analysis (IPA) of the differentially expressed genes in both clones of rMAPCs identified predominantly developmental and cardiaovascular system associated genes (table represents data obtained as output from IPA). **B**. Pathways relating to cardiogenesis were among the top canonical pathways (table represents data obtained as output from IPA). **C**. Validation by PCR of the target TF genes identified by microarray analysis.

We next evaluated changes in the global gene expression pattern during TGFβ1-mediated differentiation of rMAPCs by microarray experiments on two independent rMAPC lines, 3c3 and Cl19 (GEO number – GSE32990 and can be accessed at link provided in Supporting Information S3), in triplicate on d2, d4 and d6 of differentiation and compared the expression pattern with the expression pattern on d0. Multiple probes for a particular gene from the normalized data were averaged. Relying on the selection algorithm, previously described [22], we identified 550 probes that were differentially expressed during the differentiation in both cell lines. Of these probes, 415 have an available annotation and map to 369 different genes (Table S1). Among these 550 probes, 336 probes were also expressed in primary RAOSMCs (Table S1). Further analysis of the 550 differentially expressed probes using the Ingenuity Pathway Analysis software (IPA – Ingenuity systems, CA, USA), identified pathways involved in developmental processes including organogenesis and accompanying morphogenesis, and cellular processes including tight junction signaling among the top biological functions being represented (Fig. 2). Similarly among the canonical pathways, cardiogenesis and pathways related to human ESC pluripotency were highly represented (Fig. 2B).

We next examined the list of genes that were differentially expressed during SMC differentiation to identify candidate-regulatory transcription factors (TFs) in SMC differentiation. Transcripts for factors known to play a role in mesendoderm and mesoderm specification [27], including *Lhx1, Cited4*, *Eomes* and *Sox4*, were up regulated (Fig. 2C). TFs that maintain “stemness”/pluripotency and/or inhibit differentiation, including *Oct4*, were down regulated (Table. S1). *Foxc1/Foxc2* are known to regulate cardiovascular development [28], [29]. Although *Foxc1* transcript levels decreased during differentiation, transcript levels of *Foxc2*, which can replace *Foxc1* [29], were high (Fig. S6). Transcript levels for *Zeb1*, which inhibits collagen expression in vascular SMCs [30], were downregulated (not shown). Among the other differentially regulated TFs, the transcript levels of *Baf60c*, which is known to be important for skeletal and cardiac muscle development [31], [32], were significantly induced during differentiation. Consistent with this, we also demonstrated that *Baf60c* is expressed in primary cultured RAOSMCs (Fig. S7A).

### *Baf60c* is important for SMC generation from rMAPC

**Figure 3:**
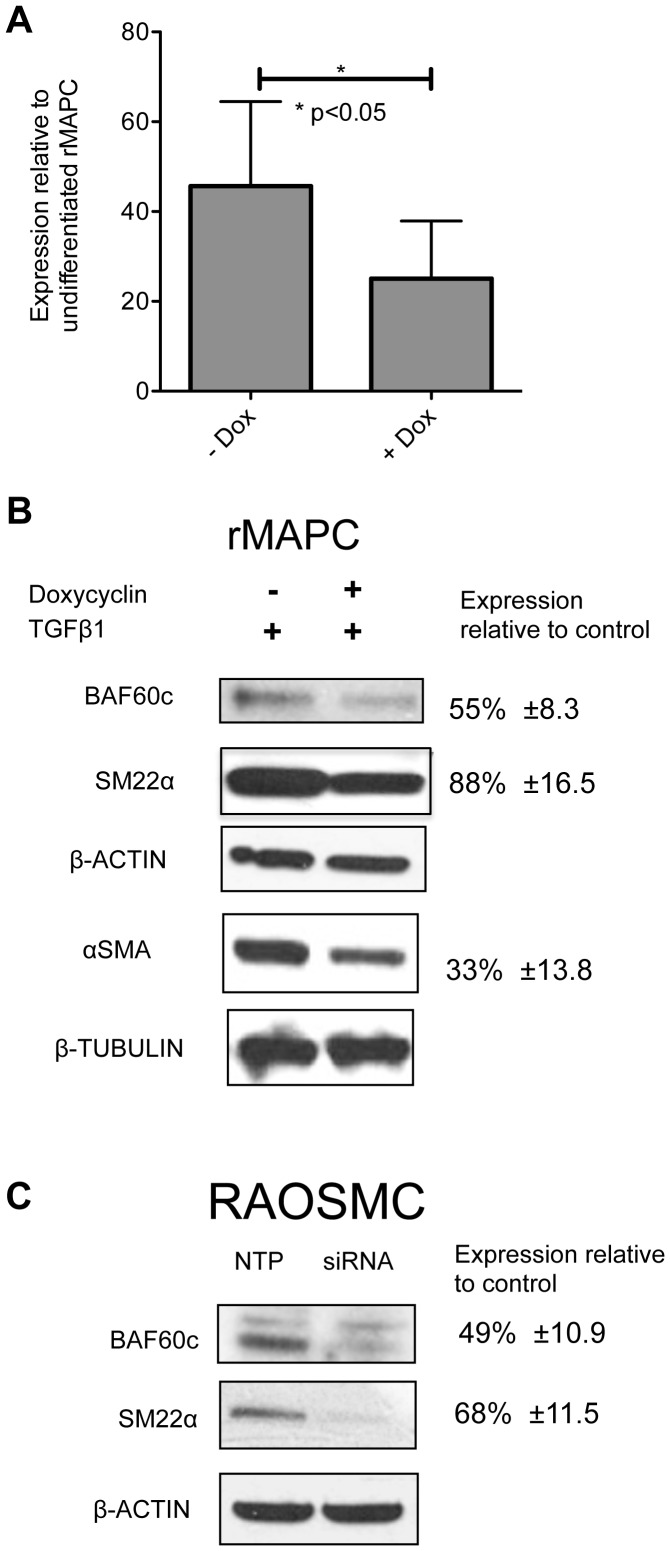
Knock down of *Baf60c* down regulates αSMA protein levels. rMAPCs were transduced with a doxycyclin (dox) inducible anti-*Baf60c* shRNA containing lentiviral vector or a vector containing a scrambled shRNA and subjected to SMC differentiation using TGFβ1. **A**. Quantitative RT-PCR for *Baf60c* mRNA levels in cells transduced with anti-*Baf60c* shRNAs in presence (+Dox) or absence (−Dox) of doxycycline up on TGFβ1 stimulation (Mean±s.e.m of n = 5; Student's t-test p<0.05). **B**. Western blots for Baf60c, αSMA, SM22α expression in cells transduced with anti-*Baf60c* shRNAs in the presence (+) or absence (−) of doxycyclin in rMAPC treated with TGF-β. **C**. Western blots for BAF60c and SM22α in RAOSMC treated with either siRNA against *Baf60c* or control.

As expression of *Baf60c* was induced strongly during rMAPC differentiation to SMCs, we tested whether *Baf60c* is responsible for the induction of SMC gene expression by performing RNAi-mediated knock down of *Baf60c*, using an inducible shRNA construct. Compared with control cells where shRNA was not induced (−Dox) or scrambled shRNA was used, levels of *Baf60c* were suppressed significantly in shRNA expressing (+Dox) cells (Figs. 3A,B, S7A). As a result of the inhibition of *Baf60c* expression, a significant decrease in αSMA (p<0.005) protein levels was seen in rMAPCs differentiated to SMC with TGFβ1 (and PDGF-BB) (Fig. 3B). As we could not generate a second *Baf60c*-targeting shRNA vector with better knock down efficiency, we also transiently transfected rMAPC with an on-target-siRNA pool against *Baf60c* and submitted the cells to SMC differentiation with TGFβ1. The percent knockdown with the on-target-siRNA pool was 52%, compared with a non-targeting pool of siRNA control cells. As we had seen for the shRNA-mediated knock down, this on-target-siRNA pool directed against *Baf60c* caused a decrease in *αSma* as well as *Sm22α* transcript levels (Fig. S7B). Intriguingly, we found an increase in the mature SMC marker transcript, *Cnn2* (Fig. S7B), and a minor increase in *Sm-mhc* transcripts (data not shown). Noteworthy, knockdown of *Baf60c* in rMAPC while inducing SMC differentiation with TGFβ1 led to a considerable degree of cell death (Fig. S7C), suggesting that only those cells still expressing some *Baf60c* survived, which may explain the rather limited *Baf60c* suppression. Despite the only 50% knock down of *Baf60c*, we detected significantly less induction of smooth muscle genes (Fig 3A,B). These studies thus suggest that BAF60c, a subunit of chromatin remodeling complexes, is important for SMC gene regulation.

**Figure 4:**
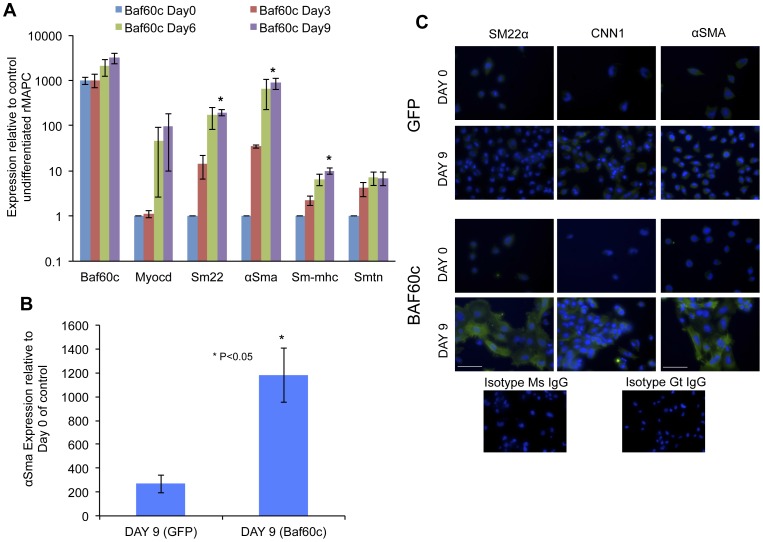
*Baf60c* is sufficient to induce smooth muscle like fate in rMAPCs. rMAPCs were transduced with an inducible *Baf60c* and eGFP cDNA encoding lentiviral vector or a vector encoding only eGFP, and subjected to SMCs without exogenous TGFβ1, and differentiation to SMCs examined by RT-qPCR and immunostaining. **A**. Expression of smooth muscle specific mRNAs compared to undifferentiated rMAPC (expression normalized to GAPDH) (Mean±s.e.m of n = 4; p<0.05). **B**. The expression of *αSma* in *Baf60c* transduced cells on day 9 cells is significantly higher compared to GFP transduced control cells (Mean±s.e.m of n = 4; Student's t-test p<0.05). **C**. *Baf60c* overexpression is sufficient to induce smooth muscle like fate with cells expressing mature smooth muscle proteins as compared to GFP transduced cells (smooth muscle proteins are colored pseudo green).

Next, we tested if BAF60c is sufficient to commit rMAPCs to the SMC lineage by forced expression of an inducible *Baf60c*-encoding construct in rMAPCs. We generated stably transformed doxycyclin-responsive clones of rMAPCs that express GFP and V5-tagged BAF60c. As control, a construct encoding only GFP was used. Following addition of doxycyclin, levels of *Baf60c* mRNA were increased by >1000-fold in the absence of TGFβ1 (data not shown). When *Baf60c*-transduced rMAPCs were cultured in serum free medium without growth factors but with addition of doxycyclin, rMAPCs differentiated to SMC (Fig. 4A) with progressive induction of transcripts and proteins for SMC structural genes (Fig. 4A,C), even though 9 days were required to attain maximal expression of SMC specific transcripts and proteins to similar levels as when differentiations were done with TGFβ1 (Figs. 4A,B,1A and S8). We noted that the frequency of cells with a more mature SMC phenotype was lower than in TGFβ1 mediated cultures (with only 30–40% cells having stress fibers) (Fig. 4B), suggesting that continued presence of *Baf60c* may prevent acquisition of a mature contractile state.

### *Baf60c* is important for phenotypic modulation of adult smooth muscle cells

**Figure 5:**
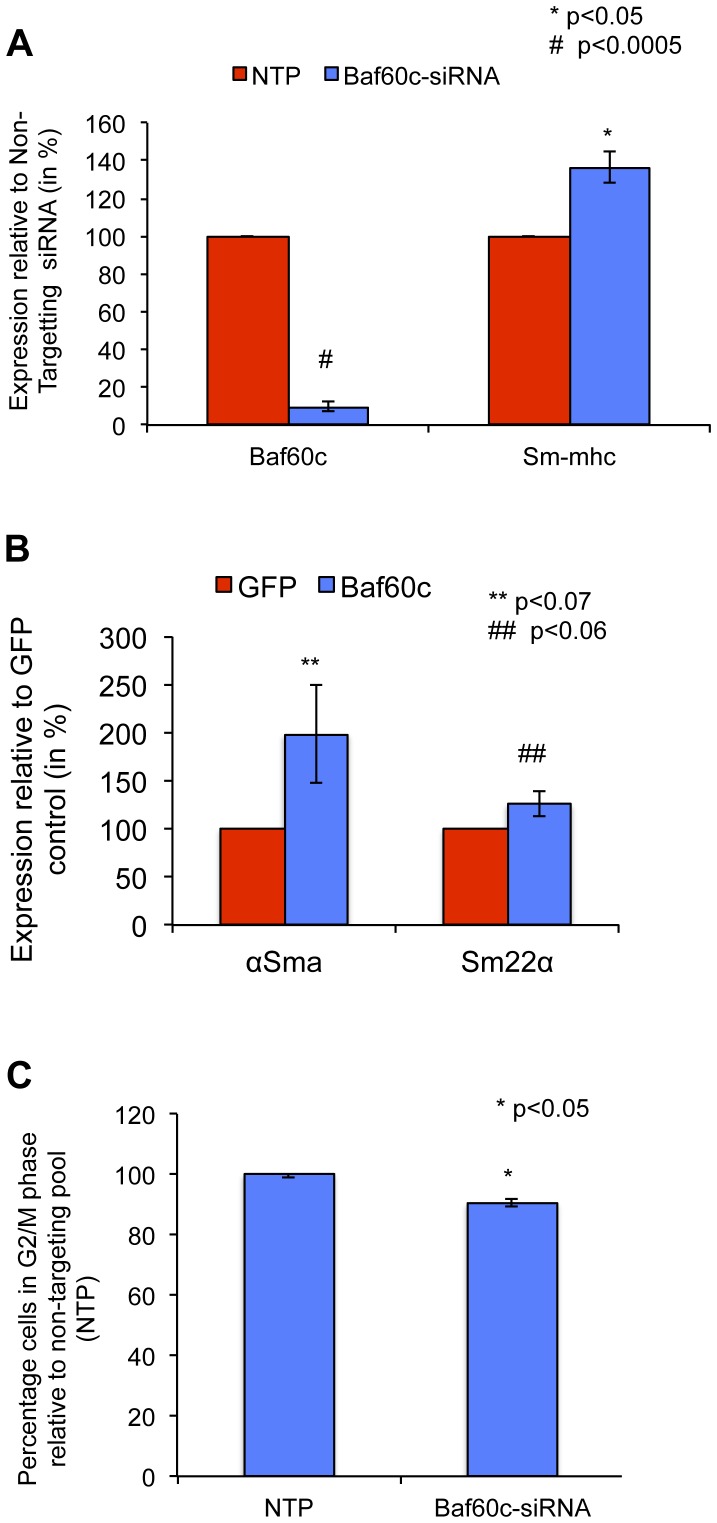
*Baf60c* is important for the synthetic/proliferative phase of primary smooth muscle cells. RAOSMCs were either transfected with an on-target anti-*Baf60c* siRNA pool vs non-targeting pool (NTP) of siRNAs; or transduced with a lentiviral vector encoding for BAF60c or GFP **A**. qRT-PCR for the expression of *Baf60c, Sm-mhc*, and α*Sma* in cells transfected with on-target anti-*Baf60c* siRNA pool vs non-targeting pool (NTP) of siRNAs (Mean ±s.e.m of n = 4; Student's t-test p<0.05). **B**. qRT-PCR for the expression of *Baf60c, Sm-mhc*, and α*Sma* in cells transduced with either GFP or BAF60c (Mean±s.e.m of n = 3–5). **C**. Cell cycle analysis using fluorescence activated cell sorting in cells transfected with on-target anti-*Baf60c* siRNA pool vs non-targeting pool (NTP) of siRNAs (Mean±s.e.m of n = 3; Student's t-test p<0.05).

We tested the effect of forced expression and knockdown of *Baf60c* in RAOSMCs. Forced expression of *Baf60c* also led to increased expression of the early smooth muscle markers such as *Sm22α* (125%, ±13.0) and *αSma* (198.5%, ±51.0) (Fig. 5B) whereas knock down of *Baf60c* in RAOSMC increased transcripts for *Sm-mhc* (136.3%±8.2) (Fig. 5A), the contractile marker of SMCs. Knock down of *Baf60c* in RAOSMC inhibited the fraction of cells in G2/M phase (90, ±1.0, p<0.05) (Figs. 5C, S9), These data suggest thus that continued expression of *Baf60c* may retain SMCs in a proliferative state and prevent maturation to a contractile state.

### *Baf60c* is direct target of SMAD-mediated TGFβ signaling

**Figure 6:**
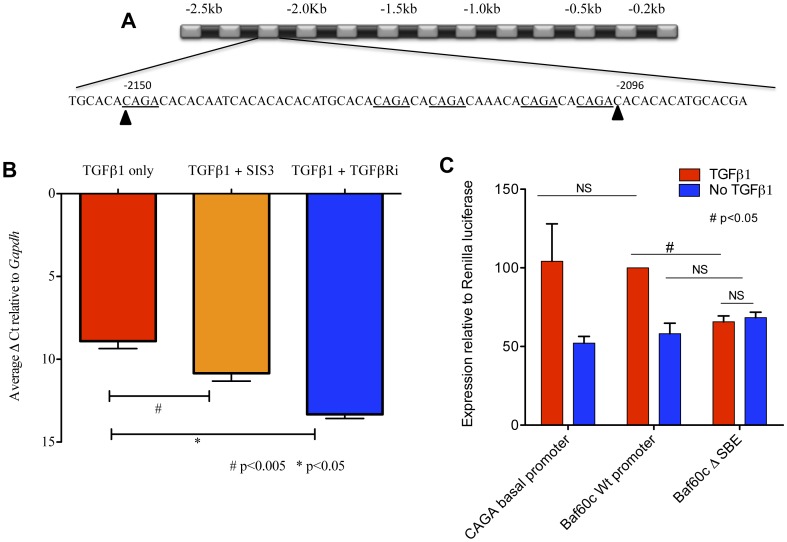
*Baf60c* is a direct target of SMAD mediated TGF-β signaling. **A**. SMAD binding elements between -2096 and -2150 bp in the *Baf60c* promoter (underlined). **B**. rMAPCs were cultured with TGFβ1 alone or combined with an inhibitor against SMAD3 phosphorylation (SIS3) or TGFβR inhibitor (TGFβRi) (SB431542) On day 2 after treatment, cells were harvested and RT-qPCR used to detect expression of *Baf60c* transcripts (Mean±s.e.m of n = 3; Student's t-test p<0.05). **C**. The intact *Baf60c* promoter (wild type) or promoter wherein the SBEs between -2096 and -2150 bp had been deleted (mutant) was cloned before a luciferase expression cassette. These constructs together with a CAGA rich promoter sequence (CAGA-control) were transfected in rMAPCs. Following addition of TGFβ1 or in absence of TGFβ1, luciferase activity was measured (Mean±s.e.m of n = 3; Student's t-test p<0.05, NS- not significant).

As *Baf60c* induced a SMC fate in rMAPCs similar to TGFβ1, we tested if *Baf60c* was a direct transcriptional target of TGFβ1 signaling. Canonical TGFβ1 signaling occurs via activation of the heterotetrameric receptors complexes composed of ALK5 and single type II receptor TGFβRII. Alk5 in this receptor complex phosphorylates the R-Smads, SMAD2 and SMAD3. Together with SMAD4, these activated Smad complexes are known to bind directly to Smad-binding elements (SBEs) composed of CAGA [33], and, mostly in concert with other and often Smad-interacting TFs, modulate expression of target genes for TGFβ. We found that the 5′ upstream region of *Baf60c* contains several such SBEs located between -2096 bp and -2150 bp (Fig. 6A).

To further test whether TGFβ1-activated signaling directly mediates *Baf60c* upregulation, we first blocked receptor activation using the ALK-4/5/7 specific inhibitor, SB431542. Addition of SB431542 to rMAPC cultures treated with TGFβ1 significantly inhibited *Baf60c* transcript upregulation compared to cells treated with TGFβ1 only (Fig. 6B). To test whether, SMAD2/3 in the TGFβ1-SMAD2/3 pathway is responsible for this upregulation, we treated rMAPCs for 48 h with either TGFβ1 alone or in combination with the SMAD3 specific inhibitor, SIS3 [34], SMAD3 phosphorylation was significantly reduced in cells treated with SIS3 (Fig. S10). Inhibition of SMAD3 phosphorylation by SIS3 also resulted in a significant decrease in *Baf60c* induction (Fig. 6B).

To further establish that TGFβ1-SMAD2/3 mediates activation of *Baf60c* expression, we performed *Baf60c* promoter-based luciferase assays. We also generated mutant vectors that lacked one or more CAGA SBEs in the *Baf60c* promoter (Fig. 6A). The CAGA-rich basal promoter served as positive control. The relative expression of luciferase from the wild-type *Baf60c* promoter fragment in rMAPCs treated with TGFβ1 or without TGFβ1 was compared to that of the mutated promoters (Fig. 6C). TGFβ1 was found to induce Luciferase expression from the wild-type (WT) promoter, and this was significantly inhibited (65.6%±3.70, p<0.05) to basal level (without TGFβ1) when the SBEs in the *Baf60c* promoter were deleted (Fig. 6A).

### BAF60c binds to the SRF binding CArG box element of smooth muscle gene promoters

**Figure 7:**
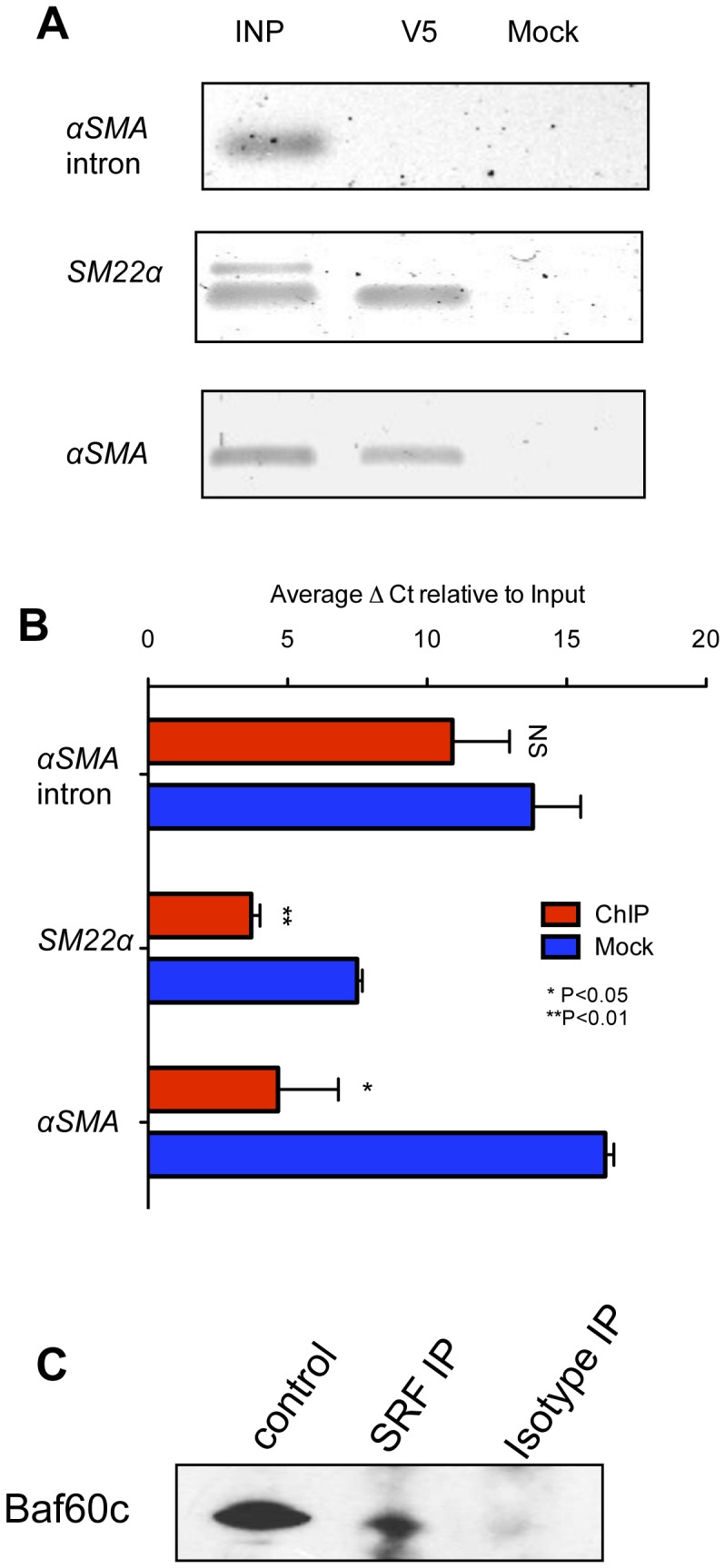
BAF60c associates with SRF in binding to DNA elements (CArG boxes) in regulating smooth muscle genes. V5 tagged BAF60c expressing clones of rMAPC were treated with TGFβ1 for 2 days. Cells were lysed, sonicated, and chromatin bound by BAF60c was immunoprecipitated with an anti-V5 antibody. The immunoprecipitated DNA was amplified using primers surrounding CArG boxes in the promoters of *Sm22α* and α*Sma*. **A**. Representative example of 3 runs on an agarose gel. **B**. Real time PCR quantification for CArG box containing sequences in the *αSma* (Mean±s.e.m of n = 3; Student's p<0.05) and *Sm22α* (Mean±s.e.m of n = 3; Student's t-test p<0.01) promoters in chromatin IP using V5 antibody compared to isotype (mock) control antibody and negative control *αSma* intron region (Mean±s.e.m of n = 3; Student's t-test p>0.05). **C**. Co-immunoprecipitation using an antibody against SRF was probed with an antibody against Baf60c. We detected a Baf60c specific band that was not detected in the IgG control. Protein extracts from 293t cells transiently transfected with Baf60c vector was used as positive control.

BAF60c is a protein participating in chromatin remodeling by forming complexes with different regulatory proteins. In embryonic development, BAF60c interacts with Notch signaling in regulating target gene expression and aids GATA-4 in inducing a cardiac fate [35], [36]. In SMC development, SRF binding to CArG boxes plays an important role (reviewed by Miano *et al.*) [37]. We hypothesized that BAF60c-mediated regulation of SMC genes might be via interaction with CArG boxes (represented in Fig. S11). To test this, we performed chromatin immunoprecipitation (ChIP) of BAF60c bound DNA and obtained enrichment of CArG binding elements [38], [39] (Fig. 7A,B) in the *αSma* (11.7 ΔΔCt ±1.86) and *Sm22α* (3.8 ΔΔCt ±0.38) upstream regulatory region compared to the mock control.

It is well established that complexes involving SRF and MYOCD regulate CArG elements. We therefore tested if BAF60c associates with these complexes. When SRF was immunoprecipitation from primary SMCs we found that BAF60c co-eluted, suggesting a physical interaction of BAF60c with the SRF complexes (Fig. 7C). These data strongly suggests that BAF60c is involved in SMC gene expression via the previously known CArG box elements by interacting with previously characterized SRF complexes.

## Discussion

Smooth muscle cells (SMCs) play a role in many disorders. Unlike other muscle cells, however, few studies have addressed the molecular mechanisms underlying SMC development. This is in part due to embryonic lethality of candidate-gene knockout models in mice, and the fact that SMCs are derived from multiple embryonic cell populations. A number of recent studies have employed stem cells to identify molecular mechanisms underlying SMC differentiation. Sinha *et al.* have demonstrated that mouse ESCs can differentiated to SMCs via TGFβ signaling[12]. Similarly, Ross *et al.* have shown that rodent MAPCs [17], [18] could be differentiated to SMC using TGFβ1 [16]. We here confirm that rMAPC lines, which express *Oct4* at levels near levels found in ESC can be robustly differentiated to cells with SMC features in response to TGFβ1 (and PDGF-BB) in serum-free conditions. Of note, when rMAPC-derived SMCs were implanted in vivo using Matrigel-based plugs, we observed extensive contribution of the injected cells to the SMC coating of blood vessels, suggesting that rMAPC-SMCs have functional properties of mature SMCs.

Sinha *et. al*, demonstrated that SMAD-mediated TGFβ signaling activates *Myocardin* (*Myocd*) [12], which aside from serum response factor (SRF) and Myocardin-related transcription factors (MRTFs), are the only known TFs involved in SMC differentiation/maturation. Shang *et al*, identified *Pitx2* as another TF for SMC differentiation [13], even though *Pitx2* was activated by stimulation with retinoic acid, not TGFβ1. To identify signaling pathways activated during SMC differentiation from rMAPCs, we used genome-wide transcriptional analysis. We demonstrated that the mesendodermal and mesodermal TFs, including *Lhx1, Cited4*, *Eomes* and *Sox4*, were up regulated, whereas *Oct4* was down regulated, and a TF known to play a role in cardiovascular development, i.e. *Foxc2*, was highly expressed. The analysis revealed also that other TFs, not previously shown to play a role in SMC differentiation were induced/up regulated during differentiation of rMAPCs to SMCs, including *Foxj1* and *Baf60c*. Interestingly, *Baf60c* was found to be highly expressed in mesodermal committed stem cells such as MSC [21], while it is not expressed in the more primitive rMAPC clones that express *Oct4*.

BAF60c was previously shown to be important in the differentiation of both cardiac and skeletal muscle [31], [32]. Takeuchi *et. al*, demonstrated that binding of BAF60c associated complex to regulatory regions of cardiac specific genes modulates cardiac myocyte specific gene expression and ultimately induces a beating myogenic fate in generally non-cardiogenic extra embryonic endodermal cells [35]. They also demonstrated that BAF60c interacts with nuclear activated Notch-derived transcription factor co-factor (NICD) during embryonic left-right axis formation [36] and that BAF60c is required for GATA4-mediated regulation of cardiac genes. Ochi *et. al*, suggested that BAF60c may interact with Brachyury (the prototype member of T family of DNA binding transcription factor) in regulating downstream targets of skeletal muscle [32].

In this study, we demonstrated that loss of *Baf60c* by about 50% prevented SMC gene induction in rMAPCs by TGFβ1. As the same shRNA or pools of siRNAs were capable of suppressing expression of *Baf60c* in mature SMC, and knockdown of *Baf60c* in differentiating rMAPCs was associated with cell death. We hypothesize that loss of *Baf60c* during SMC differentiation causes reduced viability, aside from reduced differentiation. This would be consistent with the findings that inhibition of BRG or BRM or one of their associated complex proteins is associated with decreased differentiation as well as cell death [40]; we observed a similar cell death in anti-*Baf60c* shRNA expressing cells. Furthermore, we observed that forced expression of *Baf60c* alone could commit rMAPCs to SMCs, even in the absence of exogenous TGFβ1. Of note and aside from a possible role during development we also have found a possible role for *Baf60c* in mature smooth muscle modulation. When SMC differentiation was induced by BAF60c, we noted that the fate of the SMCs was less mature than when differentiation was induced with TGFβ1, as fewer cells had stress fibers. In vivo *Baf60c* expression is restricted to developing limb bud, neural tube and cardiac tissue [31]. By contrast cultured RAOSMCs express *Baf60c* (Fig. 5). SMCs are the only terminally differentiated cells that can undergo de-differentiation to a proliferative state influenced by the local environment such as injury. This induces release of growth factors and cytokines that induce a phenotypic switch from a contractile quiescent state to a proliferative fate. This is associated with significant changes in gene pattern [41], with loss of the quiescent/contractile markers, such as *Sm-mhc*, and loss of the typical fibrous phenotype [3], [41]. We here demonstrate that knockdown of *Baf60c* in RAOSMC causes the appearance of more mature quiescent/contractile phenotype associated with increase in mature SMC markers (such as SMMHC), which is normally reduced or lost upon prolonged passaging of cells in serum, whereas forced expression of Baf60c further induces a proliferative/synthetic fate (Figs. 4, 5). How *Baf60c* contributes to this phenotypic modulation remains to be deciphered.

We further demonstrated for SMC development from stem/progenitor cells, using promoter luciferase assays, that TGFβ1 mediated phosphorylated SMAD2/3 directly activate expression of *Baf60c*, as deletion of the SBEs between -2096 bp and -2150 bp in the promoter inhibited TGFβ1 mediated luciferase expression. Serum response factor (SRF) and its co-activators, MYOCD or MRTFs bind to CArG boxes. CArG boxes have been identified in the promoters of a number of SMC genes, including *Sm22α* [42], *αSma* [43] and other genes [44], [45]. A number of studies have shown that TGFβ1 activates SMC gene expression via MYOCD- and MRTF-A-mediated binding to CArG boxes [46], [47]. Other studies have shown that aside from SRF/MYOCD and GATA6 [48] recruitment to the promoter regions of SMC-specific genes, the recruitment of components of the SWI/SNF chromatin-remodeling complex is also required [49]. We therefore hypothesized that chromatin immunoprecipitation using an anti-*Baf60c* antibody would enrich for CArG boxes. We indeed demonstrated enrichment of SRF/MYOCD regulated regions upon chromatin immunoprecipitation of V5-tagged BAF60c in rMAPC differentiated to SMC for 3 days. We further demonstrate that BAF60c physically associates with the SRF/MYOCD transcriptional machinery. These data strongly indicates a role for BAF60c in regulating SMC genes, through the interaction with already known regulators of SMC genes such as SRF/MYOCD. Previous work and the current study, hence, indicate that BAF60c may be a general modulator that collaborates with tissue specific TFs to modulate the chromatin and activate tissue specific genes [31], [36].

The rMAPC-SMC model has proven to be helpful in understanding and identifying novel TFs responsible for SMC differentiation. However, further improvement of rMAPC-SMC commitment may well be achieved via a staged differentiation by initial commitment to mesodermal precursor and followed by SMC commitment [50]. Further analysis of the transcriptome data will be of interest to characterize the role of other TFs as well as other proteins identified to be differentially expressed during SMC differentiation of rMAPCs.

## Supporting Information

Supporting Information S1 **List of primers/oligos used.**
(DOCX)

Supporting Information S2 **List of primary and secondary antibodies.**
(DOCX)

Supporting Information S3 **Additional materials and methods.**
(DOC)

Figure S1 **Plasmid map of** ***Baf60c*** **overexpression vector.**
(TIF)

Figure S2 **Smooth muscle differentiation of additional rMAPC clones.** Differentiation of *Oct4* expressing rMAPC to smooth muscle like cells. 4 clones of rMAPC were differentiated with TGFβ1 and PDGF for 6 days. RT-qPCR for expression of smooth muscle genes represented as fold induction relative to undifferentiated rMAPC (Day 0) (Mean±SEM of n = 3–5 p<0.05).
(TIF)

Figure S3 **Smooth muscle differentiation of cl-19 rMAPC represented as delta Ct.** Differentiation of *Oct4* expressing clone (cl-19) of rMAPC to smooth muscle like cell (figure 1A). Data from fig. 1A represented as delta Ct relative to house keeping gene (*Gapdh*) (scale on y axis is reversed to indicate an increase, since lower delta Ct represents higher expression). (Mean±SEM of n = 3–5 p<0.05).
(TIF)

Figure S4 **Passaging of rMAPC in serum containing media.** rMAPC were cultured with TGFβ1 and PDGF for 6 days, and then passaged in serum containing medium without growth factors. Expression of SMC genes were examined by RT-qPCR (Mean±SEM of n = 3, p<0.05).
(TIF)

Figure S5 **Matrigel plug assay – contribution of rmapc-smc to vessel coating** ***in vivo*** **.** Undifferentiated rMAPC and rMAPC-derived SMC were injected in matrigel also containing FGF2 and VEGF under the skin of nude mice. Matrigel plugs were harvested on day 21, and the number of GFP positive SMC cells enumerated. A. Macroscopic view of matriel plugs. B. The ratio of αSMA+/GFP+ co-localized vessels for undifferentiated and pre-differentiated rMAPCs in matrigel plug assay (Mean±SEM of n = 4; p<0.005).
(TIF)

Figure S6 **Expression of Foxc1/c2 during rMAPC-SMC differentiation.** Rat MAPC were induced to differentiate in serum-free medium with TGFβ1 and PDGF as described in methods. Transcripts levels for *Foxc1* decrease while those of *Foxc2* continued to be highly expressed.
(TIF)

Figure S7 **Knock-down of** ***Baf60c*** **.** **A**. Knock-down efficiency was tested in RAOSMC by transient transfection with an inducible lentiviral vector encoding for anti-*Baf60c* shRNA or a non-silencing shRNA, and expression of *Baf60c* transcripts evaluated by RT-qPCR 48 h after induction of the shRNA by Dox (shown as 70.07±4.72). **B**. rMAPC were transfected with a pool of anti-*Baf60c* siRNAs or non-targeting control (NTC), and expression of SMC specific genes evaluated after culture of the transfected cells with TGFβ1, (shown as Mean±SEM of n = 3–4; p<0.05). **C**. Presence of shRNA against Baf60c caused a significant cell death on Day 2 after induction during rMAPC-SMC differentiation (Mean±SEM of n = 3, P<0.05).
(TIF)

Figure S8 **Overexpression of** ***Baf60c*** **in rMAPC.** *Baf60c* expression was sufficient to induce smooth muscle genes in absence of exogenous TGFβ1. The expression of SMC markers was significantly higher compared to GFP expressing controls. (Mean of n =  3–5, p<0.05).
(TIF)

Figure S9 **Cell cycle analysis of RAOSMC in absence of** ***Baf60c*** **.** Knock down of Baf60c in primary smooth muscle cells leads to inhibition of proliferation compared to non-targeting pool of siRNA (Mean of n = 3, p<0.05) (representative cell cycle curve).
(TIF)

Figure S10 **Inhibition of SMAD3 phosphorylation.** rMAPC were incubated with TGFβ1 alone or combined with the SMAD3 inhibitor SIS3. Activation of SMAD3 was identified using an antibody against total as well as phosphorylated SMAD3.
(TIF)

Figure S11 **Smooth muscle promoter(s) representing CArG elements.** The regions of CArG box elements in the promoters of αSma and Sm22α. The exon 1 and TATA box are represented in αSma. Sm22 lacks a putative TATA binding element. Similarly, the CArG box elements are represented in bold and underlined.
(TIF)

Table S1 **Table – differentially regulated genes during rMAPC-SMC differentiation.**
(XLS)
