# Supplementary material for: TGFβ1-Induced Baf60c Regulates both Smooth Muscle Cell Commitment and Quiescence
Source: PLoS One. 2012 Oct 26;7(10):e47629. doi: 10.1371/journal.pone.0047629 (PMC3482188; doi:10.1371/journal.pone.0047629)
Supplement: Supporting Information S1 — List of primers/oligos used. (DOCX) [file pone.0047629.s001.docx]

**S1. A –List of primers used for RT-qPCR**

| **Primer name** |  | **Sequence (5’ – 3’)** | |  | |
| --- | --- | --- | --- | --- | --- |
|  |  |  | | | |
| GAPDH fw |  | TGCACCACCAACTGCTTAG | | | |
| GAPDH rev |  | GATGCAGGGATGATGTTC | | | |
|  |  |  |  | | |
| SM22α fw |  | CCACAAACGACCAAGCCTTTT | | | |
| SM22α rev |  | CGGCTCATGCCATAGGATG | | | |
|  |  |  |  | | |
| αSMA fw |  | CGCCATCAGGAACCTCGAGA | | | |
| αSMA rev |  | CAAAGCCCGCCTTACAGA | | | |
|  |  |  |  | | |
| Myocardin fw |  | CTGTGTGGAGTCCTCAGGTCAAACC | | | |
| Myocardin rev |  | GATGTGTTGCGGGCTCTTCAG | | | |
|  |  |  |  | | |
| Calponin fw |  | ACATCATTGGCCTACAGATG | | | |
| Calponin rev |  | CAAAGATCTGCCGCTTGGTG | | | |
|  |  |  |  | | |
| SM-MHC fw |  | CAAGAGTTCCGGCAACGCTA | | | |
| SM-MHC rev |  | TCCATCCATGAAGCCTTTGG | | | |
|  |  |  |  | | |
| Smoothelin fw |  | CCAGAGGCTCCTCTAACACTAAGAG | | | |
| Smoothelin rev |  | TTGGCTCTTGATTTTGGGTTGGCTG | | | |
|  |  |  | | | |
| Baf60c fw |  | GGACCCATCAGACCAGAAGA | | | |
| Baf60c rev |  | GAGCAGGTCTTGGACGTAGC | | | |
|  | | | | |  |
| *Fw – forward, Rev- reverse |  |  | | | |

**S1. B –List of primers used for ChIP PCR**

| **Primers for ChIP** |  | **Sequence (5’ – 3’)** |  |
| --- | --- | --- | --- |
|  |  |  |  |
| SM22α fw |  | AGGAAGGTTTTCGTGGTCCT | |
| SM22α rev |  | CCCACTCACTCCACACAGG | |
|  |  |  |  |
| αSMA fw |  | TGTTTCGAGAGCAGAGCAGA | |
| αSMA rev |  | GGCTGAATGCTGAAGGGTTA | |
|  |  |  |  |
| αSMA intron fw |  | TCAGAGGGCATGTCAGAGTG | |
| αSMA intron rev |  | GTTCCAGCGGTATACGGAGA | |
| *Fw – forward, Rev- reverse | | | |
| **S1. C –*Baf60c* shRNA sequence** | | | |

TGCTGTTGACAGTGAGCGCCCTCCTAGCATTTGAGAGGAATAGTGAAGCCACAGATGTATTCCTCTCAAATGCTAGGAGGTTGCCTACTGCCTCGGA
