# Supplementary material for: TGFβ1-Induced Baf60c Regulates both Smooth Muscle Cell Commitment and Quiescence
Source: PLoS One. 2012 Oct 26;7(10):e47629. doi: 10.1371/journal.pone.0047629 (PMC3482188; doi:10.1371/journal.pone.0047629)
Supplement: Supporting Information S2 — List of primary and secondary antibodies. (DOCX) [file pone.0047629.s002.docx]

**S2 : List of primary and secondary antibodies**

|  | **Primary antibodies** | |  | | | | |  |
| --- | --- | --- | --- | --- | --- | --- | --- | --- |
|  |  | **Concentration used** | | | | |  |  |
| **Antibody** | **Company** | **IF** | | | **WB** | |  |  |
| αSMA | Sigma-Aldrich | 1: 500 | | | 1:1000 | |  |  |
| SM22α | Abcam | 1: 500 | | | 1:500 | |  |  |
| Calponin | Sigma-Aldrich | 1: 500 | | | NA | |  |  |
| SM-MHC | Sigma-Aldrich | 1: 500 | | | NA | |  |  |
| BAF60c | Santacruz | NA | | | 1:200 | |  |  |
| GFP | Clontech | 1:50 | | |  | |  |  |
| SMAD3 | Cell Signaling |  | | | 1:500 | |  |  |
| p-SMAD3 | Acris Antibodies |  | | | 1:500 | |  |  |
| β-ACTIN | Cell Signaling | NA | | | 1:1000 | |  |  |
| β-TUBULIN | Sigma Aldrich | NA | | | 1:1000 | |  |  |
|  |  |  | | |  | |  |  |
|  | **Isotype** |  | | |  | |  |  |
|  |  |  | | |  | |  |  |
| Rabbit IgG | BD bioscience |  | | |  | |  |  |
| mouse IgG1a | Sigma-Aldrich |  | | |  | |  |  |
| Mouse IgG2a | Sigma-Aldrich |  | | |  | |  |  |
| Goat IgG | Jackson labs |  | | |  | |  |  |
|  |  |  | | |  | |  |  |
|  | **Secondary Antibodies** | | |  | |  | | |
|  |  | | |  | |  | | |
| Alexa -488 Rabbit Anti-Goat | Molecular probes | 1:500 | | |  | |  |  |
| Alexa -488 Donkey Anti-mouse | Molecular probes | 1:500 | | |  | |  |  |
| Alexa -555 Donkey Anti-mouse | Molecular probes | 1:500 | | |  | |  |  |
| Alexa -488 Donkey Anti-Rabbit | Molecular probes | 1:500 | | |  | |  |  |
| Hoechst/DAPI | Molecular probes | 1:1000 | | |  | |  |  |
| Rabbit Anti-mouse-Hrp | Dako |  | | | 1:2000 | |  |  |
| Donkey Anit-Rabbit-Hrp | Santacruz |  | | | 1:5000 | |  |  |
| Donkey Anti-Goat-Hrp | Santacruz |  | | | 1:5000 | |  |  |
|  |  |  | | |  | |  |  |
