# Supplementary material for: TGFβ1-Induced Baf60c Regulates both Smooth Muscle Cell Commitment and Quiescence
Source: PLoS One. 2012 Oct 26;7(10):e47629. doi: 10.1371/journal.pone.0047629 (PMC3482188; doi:10.1371/journal.pone.0047629)
Supplement: Supporting Information S3 — Additional materials and methods. (DOC) [file pone.0047629.s003.doc]

**ADDITIONAL MATERIALS AND METHODS**

**Microarray Processing**

As described in the main text, the cells were harvested appropriately and mRNA was isolated using the Qiagen microRNA kit (Qiagen, CA, USA). Further processing was done by microarray facility at the KULeuven as follows:

*Labeling*: RNA concentration and purity were determined spectrophotometrically using the Nanodrop ND-1000 (Nanodrop Technologies, DE, USA) and RNA integrity was assessed using a Bioanalyser 2100 (Agilent, CA, USA). 1µg of total RNA from each sample was spiked with 10 viral polyA transcript controls (Agilent) and was converted to double stranded cDNA in a reverse transcription reaction. Subsequently the sample was converted to antisense cRNA, amplified and labeled with Cyanine 3-CTP (Cy3) in an *in vitro* transcription reaction according to the manufacturer’s protocol (Agilent).

*Hybridization:* Purified and labeled cRNA (28 pmol of Cy3 labeled cRNA) was hybridised on Agilent's Whole Rat Genome 4x44K arrays followed by (manual) washing, according to the manufacturer’s procedures*.*

*Scanning*: To assess the raw probe signal intensities, arrays were scanned using the Agilent DNA MicroArray Scanner with surescan High-Resolution Technology and probe signals were quantified using Agilent’s Feature Extraction software (version 10.1.1.1)

*Normalization*: The analysis is based on the expression values as obtained from the Agilent Feature Extraction (FE) Software version 10.1.1.1 (i.e., feature gProcessedSignal for the Cy3 signal). A quantile normalization was carried out on the log2 transformed gProcessedSignal values in order to normalize the intensities between each array. After this normalization step, the signal distribution of all probes on the array will be equal for each hybridization. The normalized intensity values were used for further analysis. The total number of features on the array is 45,018, of which 43,379 represent probes which measure gene expression. The other features are Agilent controls, which were removed prior to analysis.

The raw data can be accessed at the following temporary link: <http://www.ncbi.nlm.nih.gov/geo/query/acc.cgi?token=htmzfwuewkwqadm&acc=GSE32990>

**Identification of differentially expressed probes**

The mean value for replicate probes with the same Agilent identifier on each array has been calculated. Afterwards, the three biological replicates at each time point have been averaged. In order to identify differentially expressed probes, i.e. probes whose expression profile exhibits significant expression changes over time, we relied on the selection method proposed by Di Camillo *et al.* The method calculates the area of the region bounded by the temporal profile of each probe and a control profile and compares it with the area distribution under the null hypothesis of no differential expression. In our case, the control profile for each probe was taken as a constant profile with value equal to the probe expression at the first time point (d0). In order to build the area distribution under the null hypothesis, an empirical approach is followed in which 50,000 noisy profiles are built by randomly sampling from the distribution of differences between biological replicates and their area is calculated. The 99^th^ percentile of the null hypothesis area distribution is then employed as threshold to identify differentially expressed profiles with a significance of 0.01.

**Matrigel plug assay**

Matrigel plug assay was performed as previously described , 8-week old athymic nude Foxn1 mice (Jackson Laboratory, Bar Harbor, Maine, USA) were injected subcutaneously on the dorsal side with 1x10^6^ cells re-suspended in 300µl cold growth factor-reduced Matrigel supplemented with 300ng/ml recombinant human VEGF165 and 700ng/ml recombinant human-bFGF (R&D Systems). Matrigel was either mixed with GFP-labeled undifferentiated rMAPC or pre-differentiated rMAPC-SMCs and as a control, PBS, instead of cells, was added. After 3 weeks, mice were sacrificed for histological analysis of the Matrigel implants. Matrigel plugs were removed, photographed using a Zeiss Lumar dissection microscope and fixed overnight in zinc-paraformaldehyde. After rinsing, Matrigel plugs were processed for paraffin embedding. The plugs were then sectioned into 7μm sections. The de-paraffinized sections were rehydrated and stained with anti GFP antibody (to identify the eGFP-labeled rat cells). Antigen retrieval was done by incubation with trypsin and the GFP signal was revealed after amplification using the TSA-fluorescein kit. Furthermore, these sections were secondarily stained with anti-αSMA antibody to identify the smooth muscle cells. Immunofluorescence images for the entire section were taken as a mosaic and stitched to one image using an AxioImagerZ1 and associated Axiovision ver 4.8 software (both Zeiss). The entire section was analyzed and total number of vessels marked by αSMA was manually counted. Similarly total vessels marked by GFP+ and SMA+ double positive cells was counted. The ratio of double positive (GFP and SMA co-localized) to the total number of vessels was computed. Co-localized vessels per unit area of the section were computed to normalize the data for comparison between the two conditions (undifferentiated and pre-differentiated).

**References**:

1. Di Camillo B, Toffolo G, Nair SK, Greenlund LJ, Cobelli C (2007) Significance analysis of microarray transcript levels in time series experiments. BMC Bioinformatics 8 Suppl 1: S10.

2. Aranguren XL, Luttun A, Clavel C, Moreno C, Abizanda G, et al. (2007) In vitro and in vivo arterial differentiation of human multipotent adult progenitor cells. Blood 109: 2634-2642.

3. Roobrouck VD, Clavel C, Jacobs SA, Ulloa-Montoya F, Crippa S, et al. (2011) Differentiation potential of human postnatal mesenchymal stem cells, mesoangioblasts, and multipotent adult progenitor cells reflected in their transcriptome and partially influenced by the culture conditions. Stem Cells 29: 871-882.
