# Supplementary figures and images for: TGFβ1-Induced Baf60c Regulates both Smooth Muscle Cell Commitment and Quiescence
Source: PLoS One. 2012 Oct 26;7(10):e47629. doi: 10.1371/journal.pone.0047629 (PMC3482188; doi:10.1371/journal.pone.0047629)

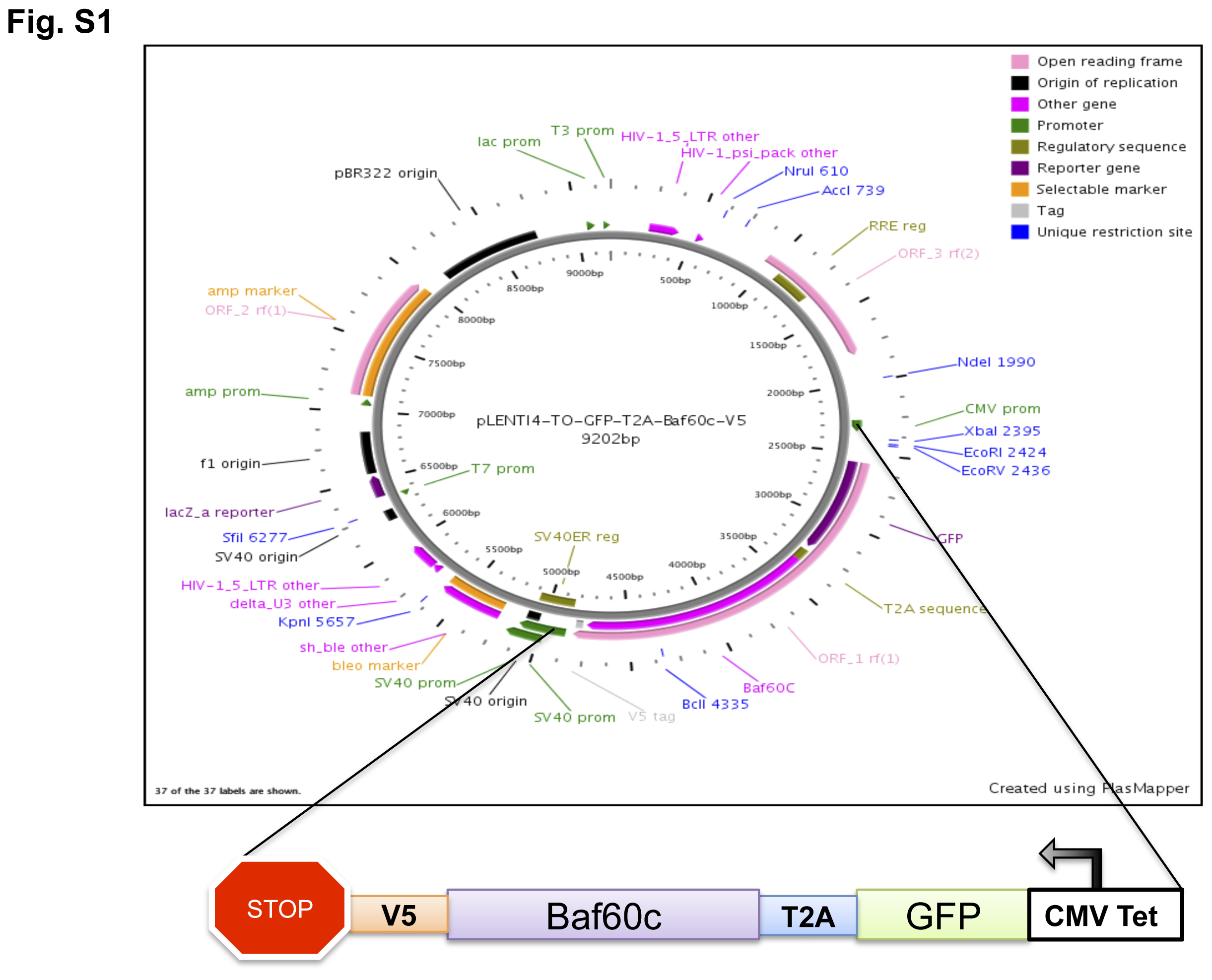

Supplement: Figure S1 — Plasmid map of Baf60c overexpression vector. (TIF) [file pone.0047629.s004.tif]

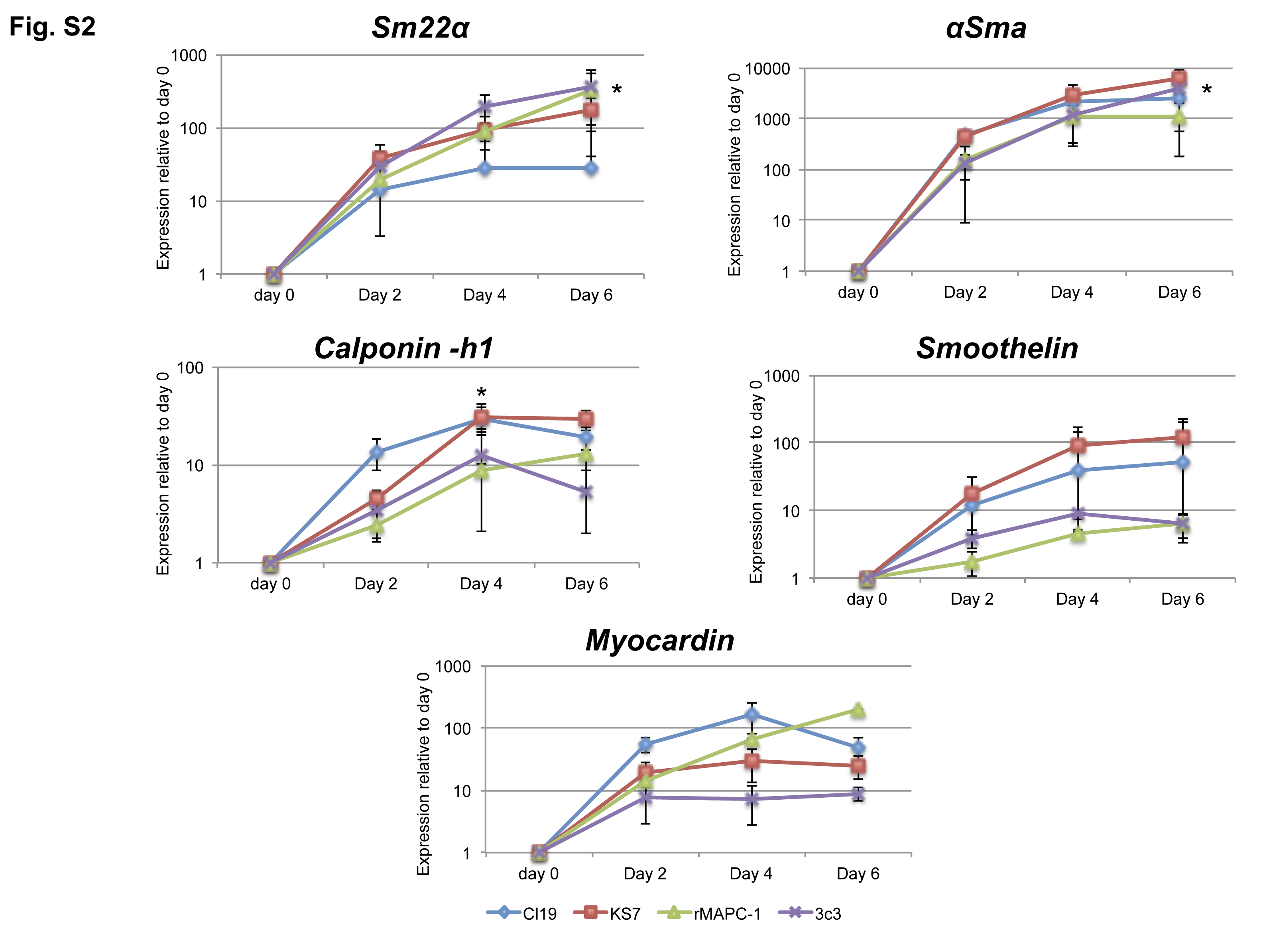

Supplement: Figure S2 — Smooth muscle differentiation of additional rMAPC clones. Differentiation of Oct4 expressing rMAPC to smooth muscle like cells. 4 clones of rMAPC were differentiated with TGFβ1 and PDGF for 6 days. RT-qPCR for expression of smooth muscle genes represented as fold induction relative to undifferentiated rMAPC (Day 0) (Mean±SEM of n = 3–5 p<0.05). (TIF) [file pone.0047629.s005.tif]

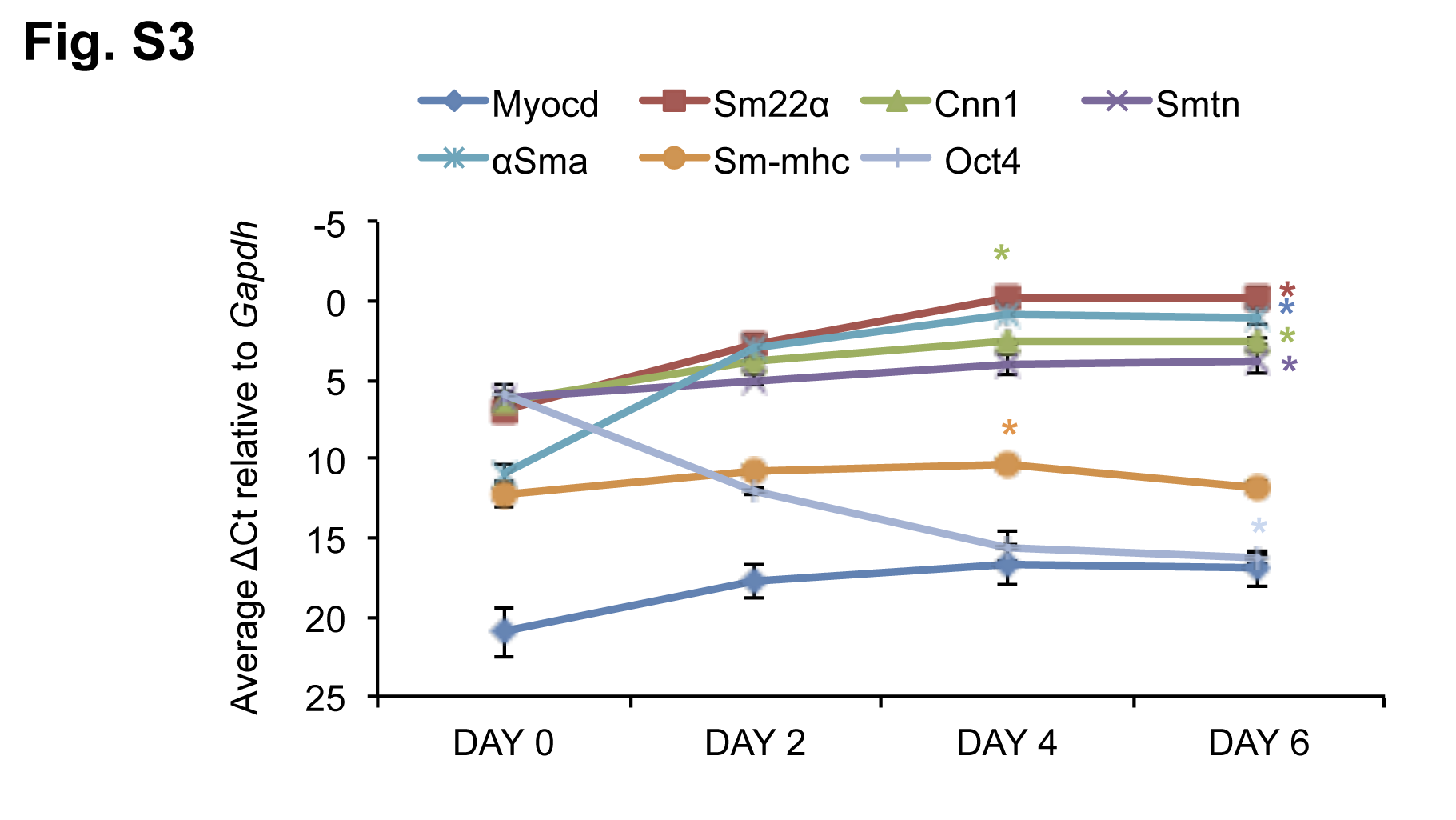

Supplement: Figure S3 — Smooth muscle differentiation of cl-19 rMAPC represented as delta Ct. Differentiation of Oct4 expressing clone (cl-19) of rMAPC to smooth muscle like cell (figure 1A). Data from fig. 1A represented as delta Ct relative to house keeping gene (Gapdh) (scale on y axis is reversed to indicate an increase, since lower delta Ct represents higher expression). (Mean±SEM of n = 3–5 p<0.05). (TIF) [file pone.0047629.s006.tif]

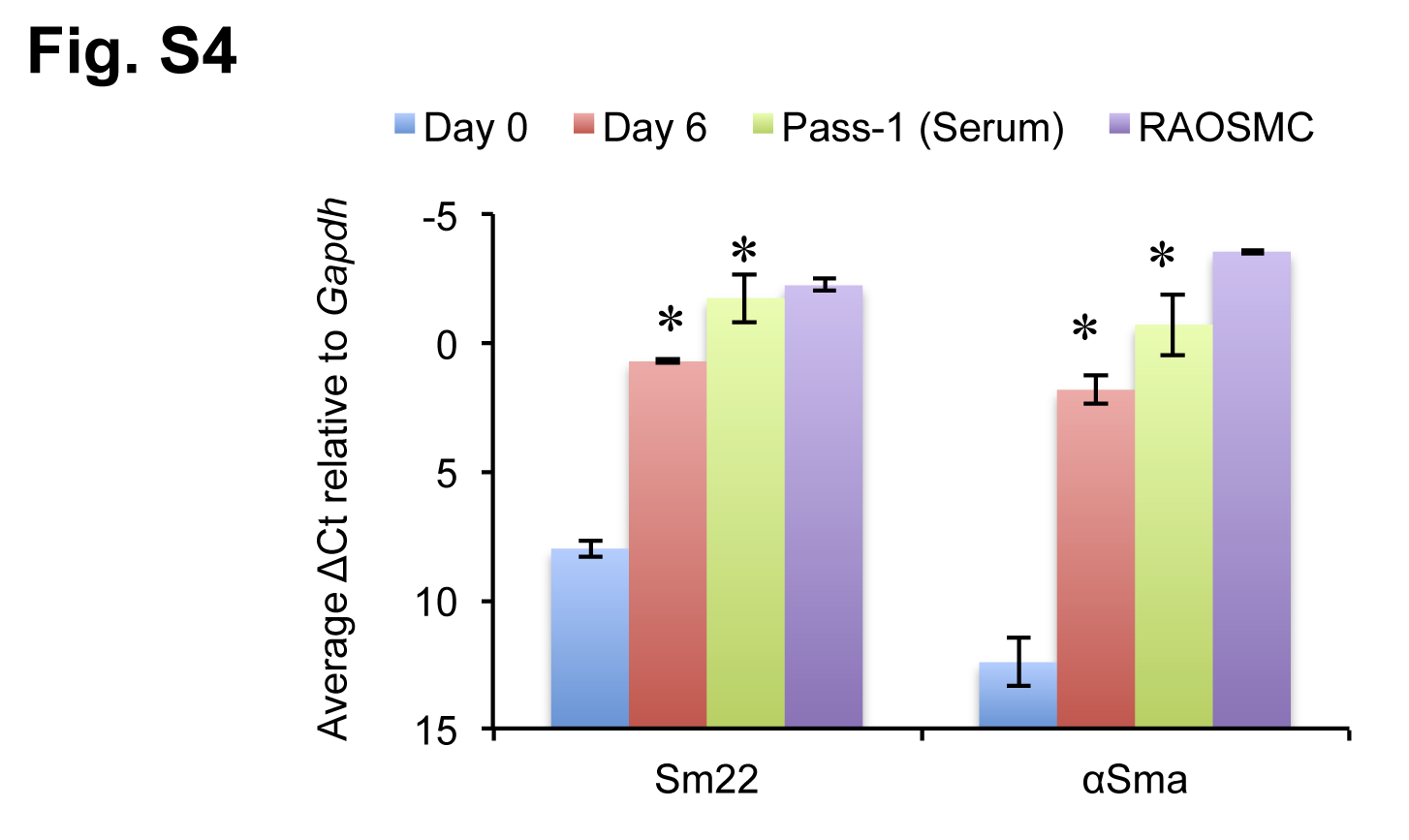

Supplement: Figure S4 — Passaging of rMAPC in serum containing media. rMAPC were cultured with TGFβ1 and PDGF for 6 days, and then passaged in serum containing medium without growth factors. Expression of SMC genes were examined by RT-qPCR (Mean±SEM of n = 3, p<0.05). (TIF) [file pone.0047629.s007.tif]

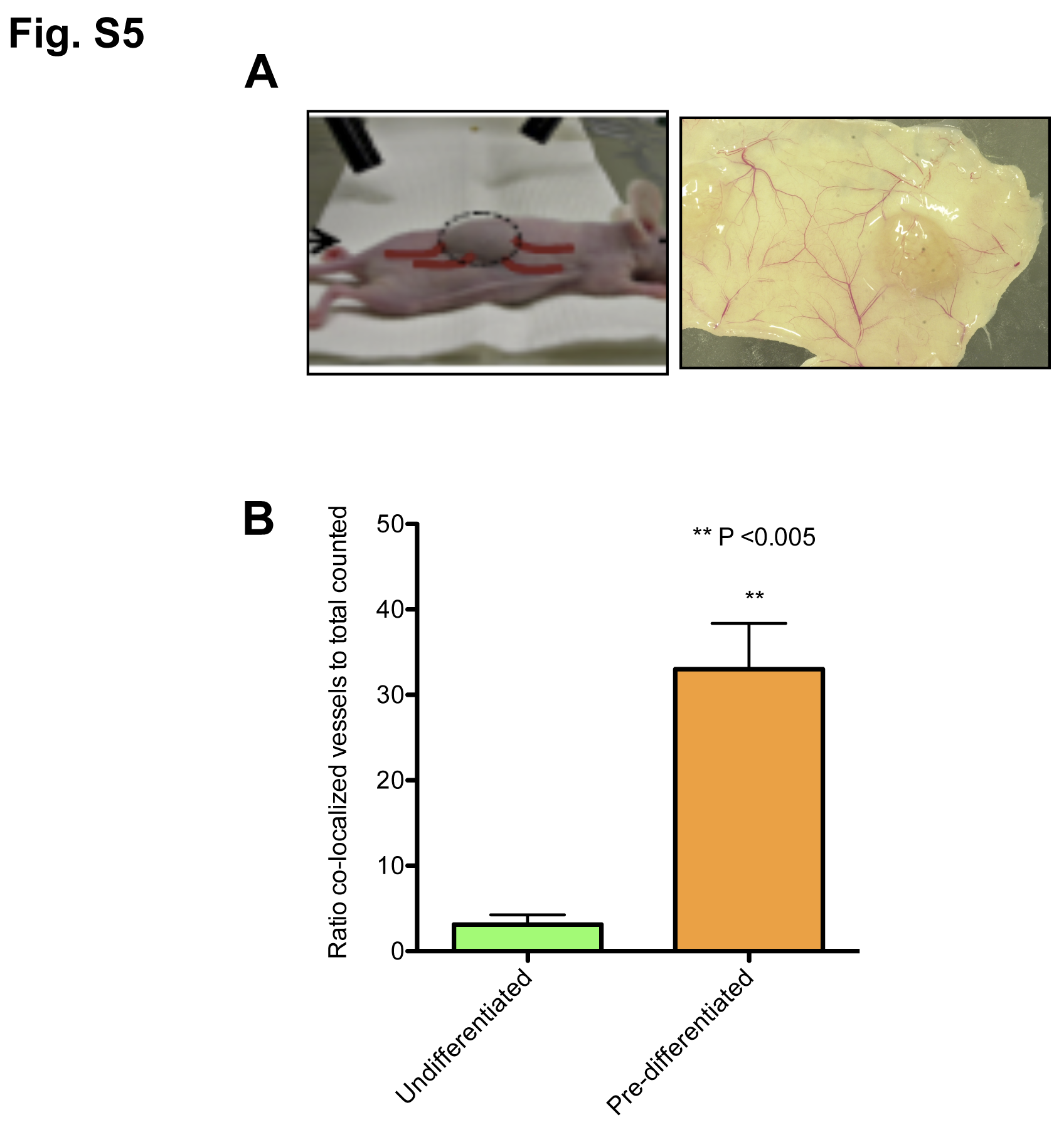

Supplement: Figure S5 — Matrigel plug assay – contribution of rmapc-smc to vessel coating in vivo . Undifferentiated rMAPC and rMAPC-derived SMC were injected in matrigel also containing FGF2 and VEGF under the skin of nude mice. Matrigel plugs were harvested on day 21, and the number of GFP positive SMC cells enumerated. A. Macroscopic view of matriel plugs. B. The ratio of αSMA+/GFP+ co-localized vessels for undifferentiated and pre-differentiated rMAPCs in matrigel plug assay (Mean±SEM of n = 4; p<0.005). (TIF) [file pone.0047629.s008.tif]

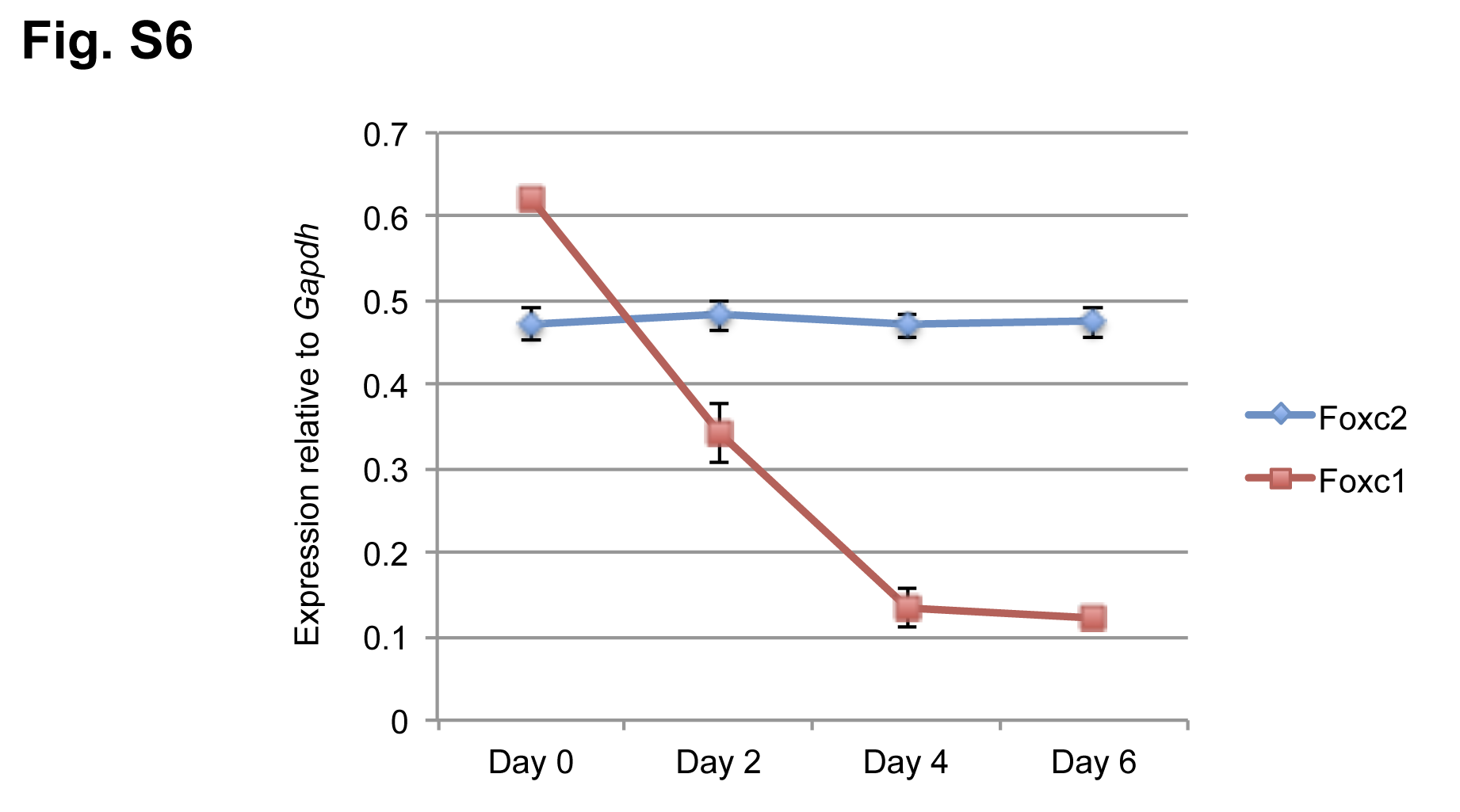

Supplement: Figure S6 — Expression of Foxc1/c2 during rMAPC-SMC differentiation. Rat MAPC were induced to differentiate in serum-free medium with TGFβ1 and PDGF as described in methods. Transcripts levels for Foxc1 decrease while those of Foxc2 continued to be highly expressed. (TIF) [file pone.0047629.s009.tif]

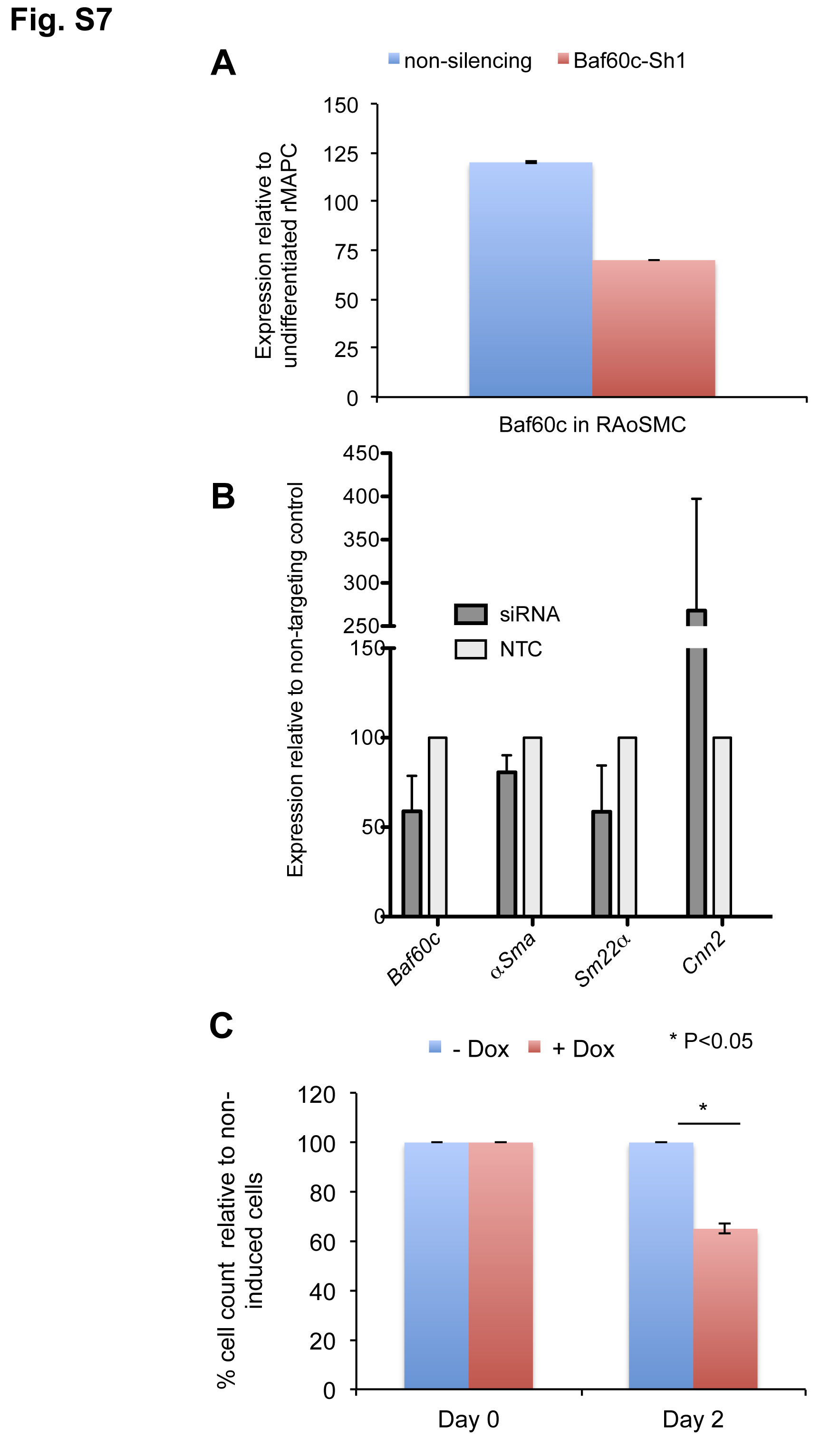

Supplement: Figure S7 — Knock-down of Baf60c . A. Knock-down efficiency was tested in RAOSMC by transient transfection with an inducible lentiviral vector encoding for anti-Baf60c shRNA or a non-silencing shRNA, and expression of Baf60c transcripts evaluated by RT-qPCR 48 h after induction of the shRNA by Dox (shown as 70.07±4.72). B. rMAPC were transfected with a pool of anti-Baf60c siRNAs or non-targeting control (NTC), and expression of SMC specific genes evaluated after culture of the transfected cells with TGFβ1, (shown as Mean±SEM of n = 3–4; p<0.05). C. Presence of shRNA against Baf60c caused a significant cell death on Day 2 after induction during rMAPC-SMC differentiation (Mean±SEM of n = 3, P<0.05). (TIF) [file pone.0047629.s010.tif]

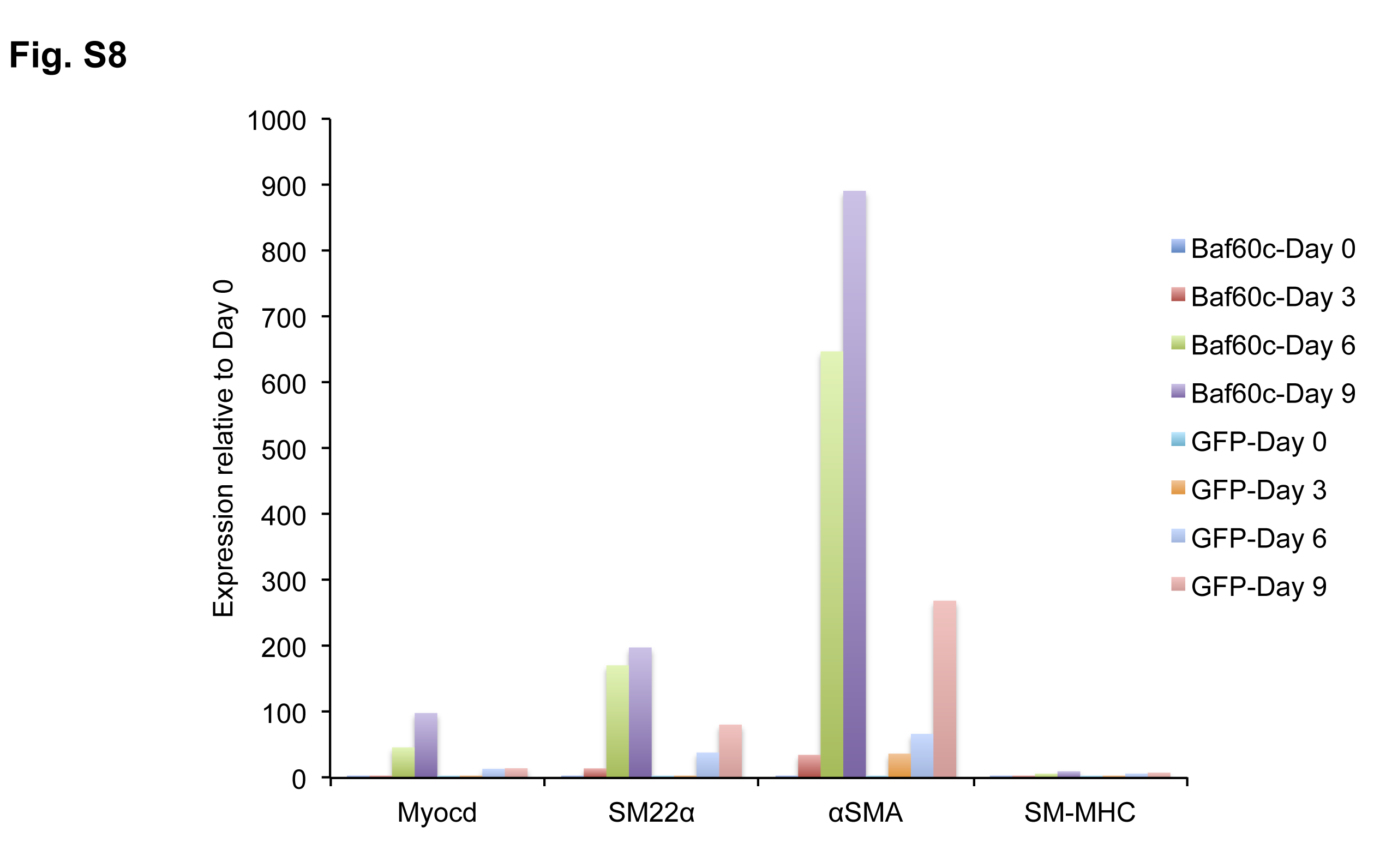

Supplement: Figure S8 — Overexpression of Baf60c in rMAPC. Baf60c expression was sufficient to induce smooth muscle genes in absence of exogenous TGFβ1. The expression of SMC markers was significantly higher compared to GFP expressing controls. (Mean of n = 3–5, p<0.05). (TIF) [file pone.0047629.s011.tif]

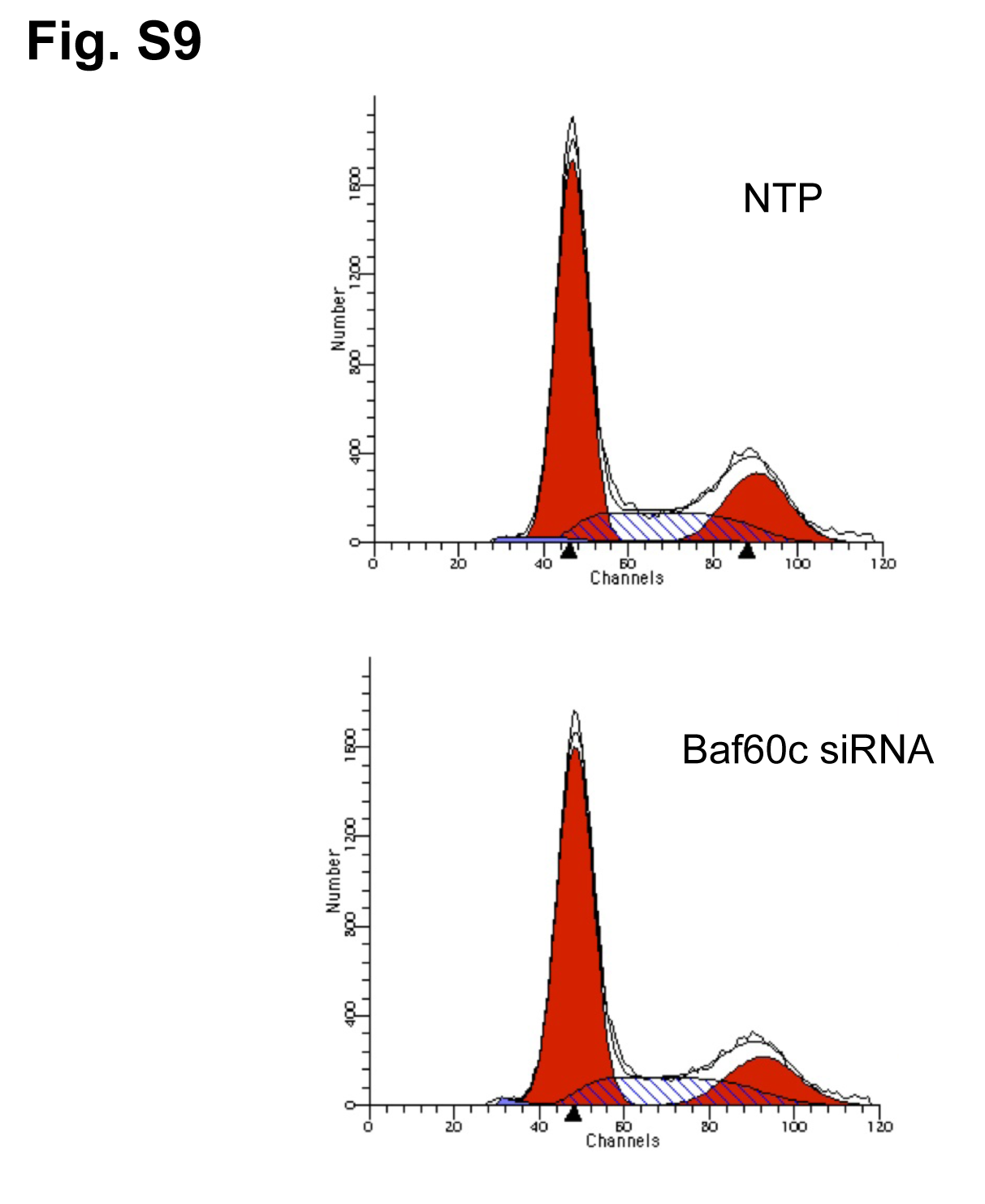

Supplement: Figure S9 — Cell cycle analysis of RAOSMC in absence of Baf60c . Knock down of Baf60c in primary smooth muscle cells leads to inhibition of proliferation compared to non-targeting pool of siRNA (Mean of n = 3, p<0.05) (representative cell cycle curve). (TIF) [file pone.0047629.s012.tif]

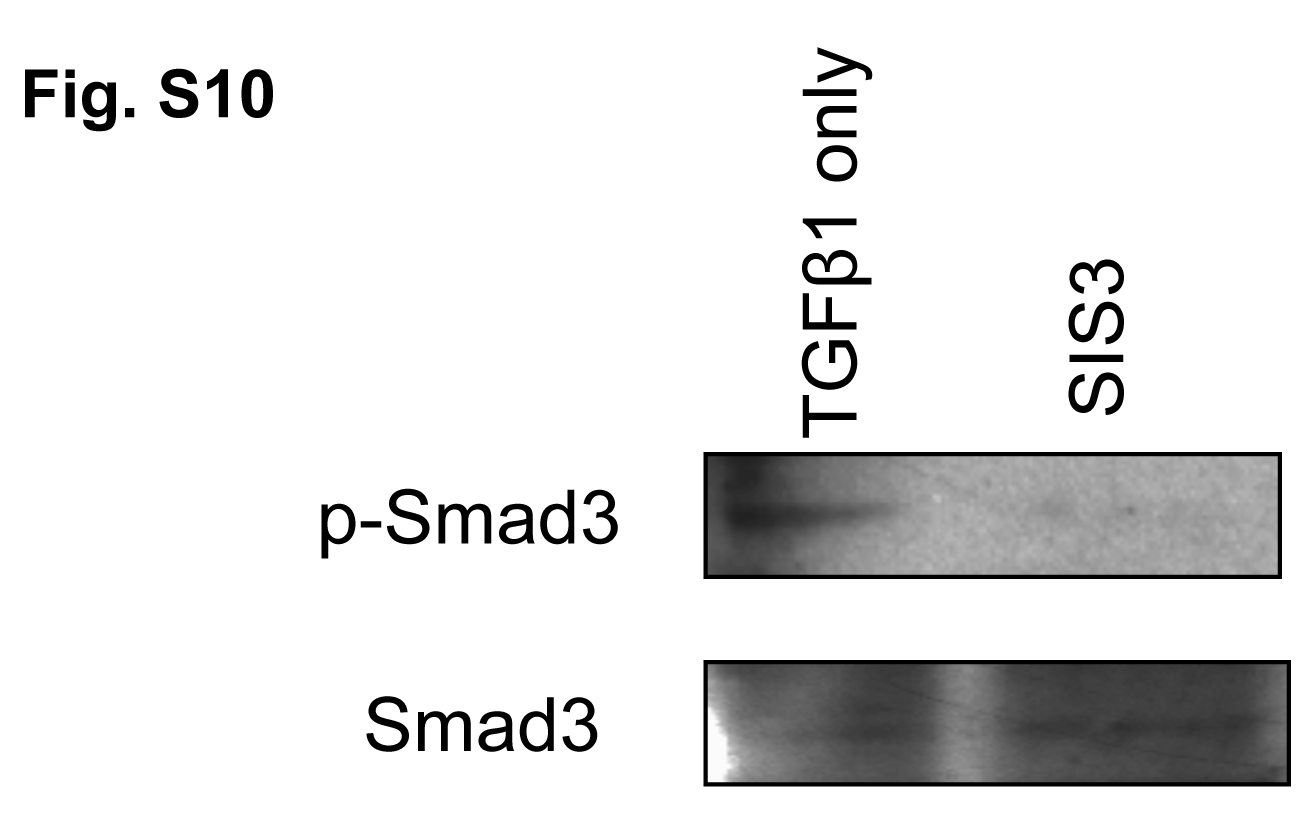

Supplement: Figure S10 — Inhibition of SMAD3 phosphorylation. rMAPC were incubated with TGFβ1 alone or combined with the SMAD3 inhibitor SIS3. Activation of SMAD3 was identified using an antibody against total as well as phosphorylated SMAD3. (TIF) [file pone.0047629.s013.tif]

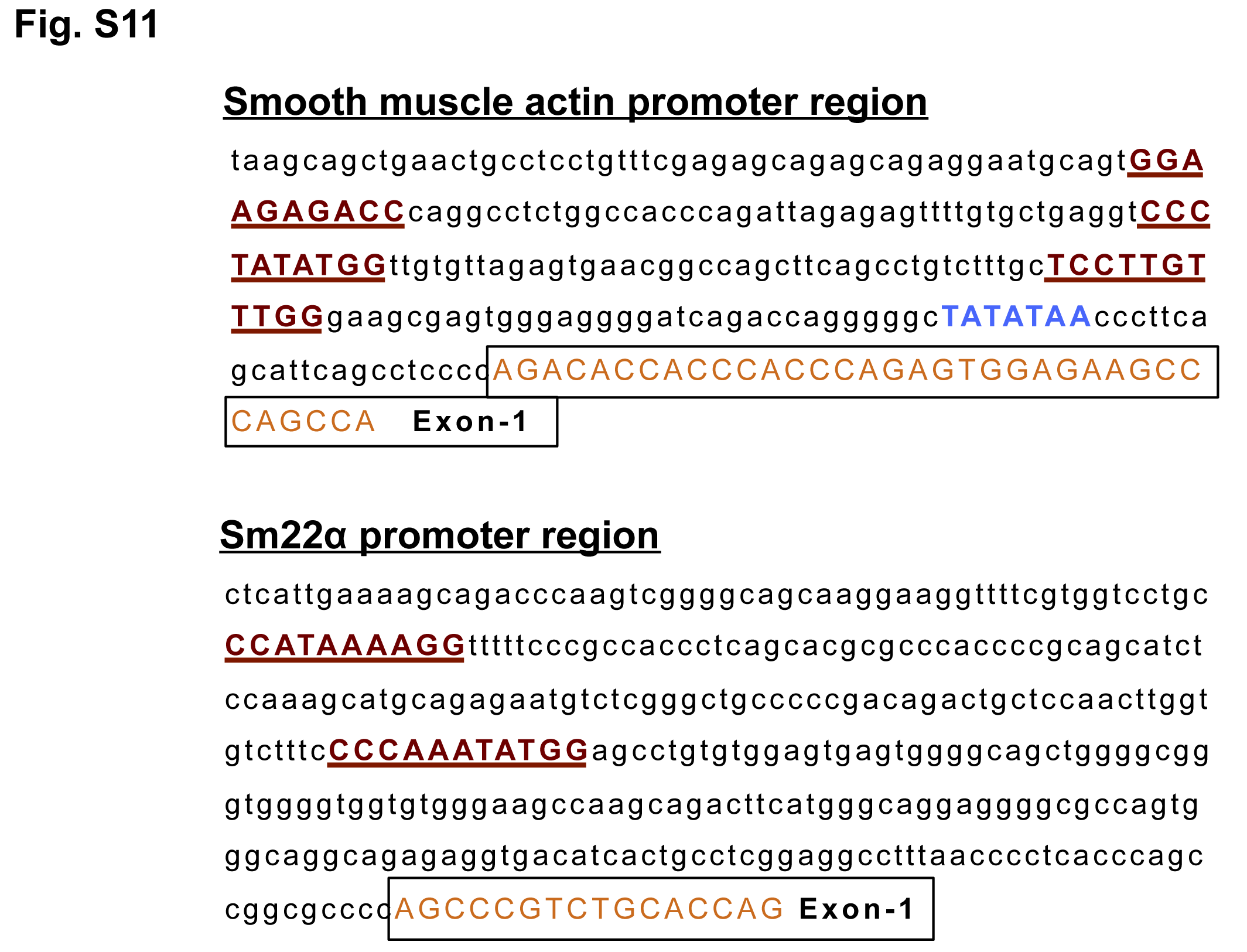

Supplement: Figure S11 — Smooth muscle promoter(s) representing CArG elements. The regions of CArG box elements in the promoters of αSma and Sm22α. The exon 1 and TATA box are represented in αSma. Sm22 lacks a putative TATA binding element. Similarly, the CArG box elements are represented in bold and underlined. (TIF) [file pone.0047629.s014.tif]
